# Wdr5-mediated H3K4me3 coordinately regulates cell differentiation, proliferation termination, and survival in digestive organogenesis

**DOI:** 10.1038/s41420-023-01529-4

**Published:** 2023-07-05

**Authors:** Zhe Zhang, Chun Yang, Zixu Wang, Liwei Guo, Yongpan Xu, Ce Gao, Yonghua Sun, Zhenhai Zhang, Jinrong Peng, Minjie Hu, Li Jan Lo, Zhipeng Ma, Jun Chen

**Affiliations:** 1 MOE Key Laboratory of Biosystems Homeostasis & Protection, College of Life Sciences, Zhejiang University, Hangzhou, 310058 China; 2 College of Animal Sciences, Zhejiang University, Hangzhou, 310058 China; 3 State Key Laboratory of Freshwater Ecology and Biotechnology, Institute of Hydrobiology, Chinese Academy of Sciences, Wuhan, 430072 China; 4 Center for Precision Medicine, Guangdong Provincial People’s Hospital, Guangdong Academy of Medical Sciences, Guangzhou, 510080 China; 5 Cancer Center, Zhejiang University, Hangzhou, 310058 China; 6 Department of Plastic Surgery, Sir Run Run Shaw Hospital, Zhejiang University School of Medicine, No. 3 Qingchun Road East, Hangzhou, 310016 China

**Keywords:** Developmental biology, Cell biology

## Abstract

Food digestion requires the cooperation of different digestive organs. The differentiation of digestive organs is crucial for larvae to start feeding. Therefore, during digestive organogenesis, cell identity and the tissue morphogenesis must be tightly coordinated but how this is accomplished is poorly understood. Here, we demonstrate that WD repeat domain 5 (Wdr5)-mediated H3K4 tri-methylation (H3K4me3) coordinately regulates cell differentiation, proliferation and apoptosis in zebrafish organogenesis of three major digestive organs including intestine, liver, and exocrine pancreas. During zebrafish digestive organogenesis, some of cells in these organ primordia usually undergo differentiation without apoptotic activity and gradually reduce their proliferation capacity. In contrast, cells in the three digestive organs of *wdr5*^*−/−*^ mutant embryos retain progenitor-like status with high proliferation rates, and undergo apoptosis. Wdr5 is a core member of COMPASS complex to implement H3K4me3 and its expression is enriched in digestive organs from 2 days post-fertilization (dpf). Further analysis reveals that lack of differentiation gene expression is due to significant decreases of H3K4me3 around the transcriptional start sites of these genes; this histone modification also reduces the proliferation capacity in differentiated cells by increasing the expression of *apc* to promote the degradation of β-Catenin; in addition, H3K4me3 promotes the expression of anti-apoptotic genes such as *xiap-like*, which modulates p53 activity to guarantee differentiated cell survival. Thus, our findings have discovered a common molecular mechanism for cell fate determination in different digestive organs during organogenesis, and also provided insights to understand mechanistic basis of human diseases in these digestive organs.

## Introduction

The vertebrate digestive track and associated organs (pancreas, liver and gall bladder) develop from a primitive gut tube that is derived from definitive endoderm [1–3]. In zebrafish, after gastrulation, a sparse layer of endoderm cells migrate medially and form a solid rod along the midline at 20 hours post-fertilization (hpf). A clear lumen can be observed in most of the gut by 42 hpf, whereas the liver and pancreatic buds are clearly identifiable at the fore-part of the rod at 50 hpf [1, 4–7]. After organ bud formation, some of cells start to differentiate. At 5 days post-fertilization (dpf), zebrafish larvae begin to feed, which requires the cooperation of different digestive organs. Therefore, it is necessary for these organ buds to coordinately develop a functional digestive system on time. It has been identified that many signal molecules (FGF, BMP, Wnt, retinoic acid (RA), Hedgehog, TGF-β and Notch, etc) [8], and transcription regulators (Gata4/5/6, FoxA1/2/3, Cdx2, Sox9, Hnf1α/β, Hnf4α, Neurogenin3, Nkx2.2, NeuroD, Pax4/6, Pdx1, Ptf1a, Hnf6, OC2, Yap1 and C/EBPα, etc) play multiple stage-specific roles in the process of different digestive organ differentiation [9], whereas some other components such as Def, Npo, Elys, Lhr-1, Sec13 and Bms1, etc, function as a pan-digestive factor to regulate the cell proliferation in the entire digestive system [8, 10–14]. However, it is not known if there is a mechanism to coordinately regulate cell differentiation in different digestive organs during organogenesis. It is also unclear how differentiated cells gradually lose their proliferation capacity and remain survival, which is one of fundamental questions in organ development.

WD repeat domain 5 (Wdr5) is best characterized as an adaptor protein of the COMPASS complex that catalyze Histone3 lysine 4 di- and tri-methylation (H3K4me2,3) [15, 16], but emerging evidence demonstrates that it has functions outside this complex including as a component of the NSL (non-specific lethal complex) for H4 lysine 16 acetylation (H4K16ac) [15, 17, 18], interacting with Oct4, Myc, p53 or lncRNAs to facilitate their target gene expression, controlling expression of genes related to protein synthesis, re-initiating transcription upon exit from mitosis, and promoting faithful assembly of mitotic spindle [19–25]. Wdr5 plays different roles in many developmental processes, for example, promoting proper bone formation and regulating self-renewal of embryonic stem cells [26]. Knockdown of Wdr5 in Xenopus leads to somitic, gut and hematopoietic developmental defects [16]. However, loss-of-function of *Wdr5* in mouse leads to heterozygous lethality at early embryonic stages before organogenesis [27]. It is difficult to pinpoint a clear biologic role for Wdr5 in organogenesis of vertebrates without a Wdr5 knockout model.

We previously demonstrated that Wdr5-mediated H3K4me3 is required for genetic compensation response [28]. Knockout of *wdr5*^*−/−*^ not only impaired genetic compensation response, but also resulted in severe developmental defects such as: curved body, undeveloped liver, small eyes, small head and dead at 7 dpf. As zebrafish *wdr5* is a maternal gene, the gastrulation of *wdr5*^*−/−*^ mutant embryos is relatively normal, which provides a good model to study *wdr5* functions in organogenesis. In this report, taking the advantage of zebrafish *wdr5*^*−/−*^ mutant, we investigate the functions of *wdr5* in digestive organogenesis.

## Results

### Wdr5 plays an essential role in the differentiation of intestine, liver, and exocrine-pancreas

**Fig. 1:**
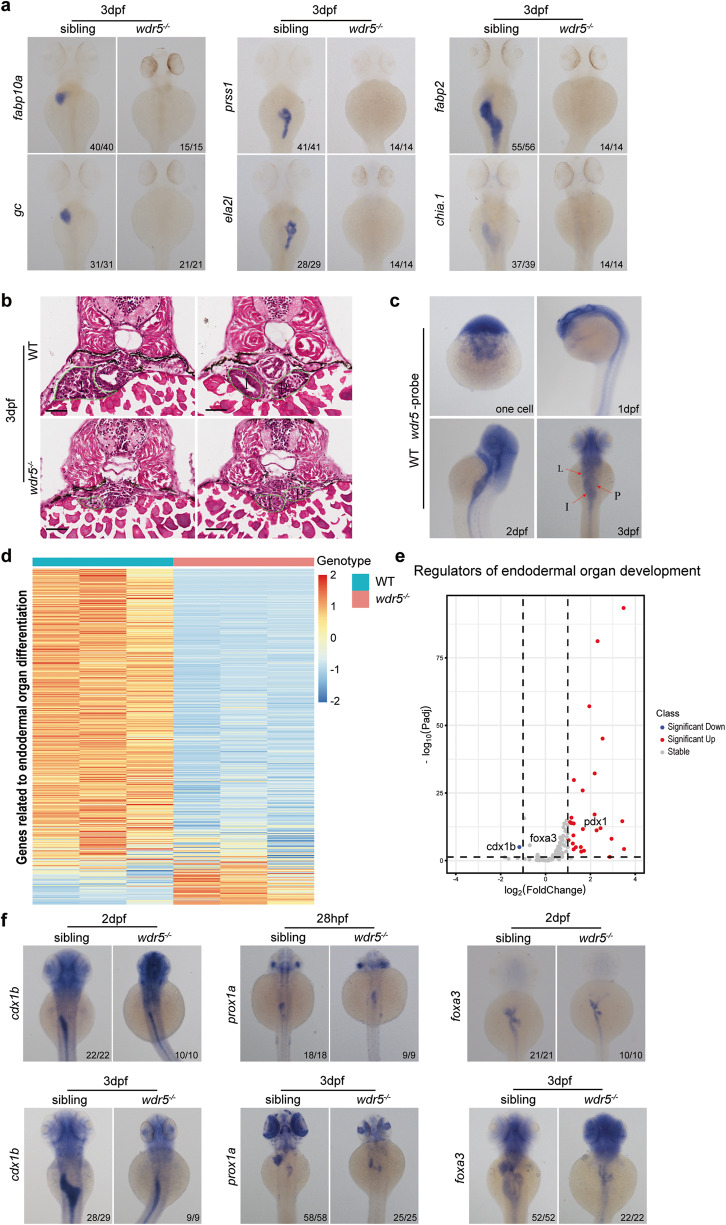
The differentiation of intestine, liver, and exocrine-pancreas is impaired in *wdr5*^−*/−*^ mutant embryos. **a** Whole-mount in situ hybridization (WISH) of wild type (WT) and *wdr5*^−*/−*^ mutant embryos at 3 dpf to show the expression of differentiation genes for different digestive organs including: the *fabp10a* and *gc* for liver, *prss1*and *ela2l* for exocrine pancreas, *fabp2* and *chia.1* for intestine as indicated. **b** Hematoxylin-Eosin (H&E) staining of cryosections to show liver (L), intestine (I) and exocrine pancreas (P) in WT and *wdr5*^*−/−*^ mutant embryos at 3 dpf. Scale bar: 40 μm. **c** WISH of *wdr5* in WT at one cell stage, 1, 2 and 3 dpf. **d** Heatmap of genes related to endodermal organ differentiation in WT and *wdr5*^*−/−*^ mutant embryos at 3 dpf. **e** Volcano plot to show differential expressed regulators of endodermal organ development between WT and *wdr5*^−*/−*^ mutant embryos at 3 dpf (*wdr5*^*−/−*^ mutant vs WT, |log_2_FoldChange | ≥ 1, *P*_*adj*_ < 0.05). **f** WISH of WT and *wdr5*^*−/−*^ mutant embryos at different time points with different digestive organ development regulators including: pan-endodermal marker *foxa3*, hepatic marker *prox1a* and intestine marker *cdx1b* as indicated. *n* indicates the number of zebrafish embryos in each group.

We previously found that the expression of *fabp10a*, a liver differentiation marker gene, was not detected in *wdr5*^*−/−*^ zebrafish mutant at 3.5 dpf [28], which raises a question of if *wdr5* functions in the development of liver or endoderm-derived organs. Interestingly, whole-mount in situ hybridization (WISH) showed that the expression of other digestive organ differentiation marker genes such as: *gc*, *fads2* of liver; *prss1*, *ela2l*, *ctrb1* of exocrine pancreas; and *fabp2*, *chia.1*, *cdh17*, *slc6a19a.1* of intestine, was also not detected in *wdr5*^*−/−*^ mutant at 3 or 5 dpf (Fig. 1a and Supplementary Fig. 1d, f), whereas cross-section showed that the primordia of liver, exocrine-pancreas and intestine were observed in *wdr5*^−*/−*^ mutant at 3 and 5 dpf, though the sizes of the *wdr5*^*−/*−^ mutant digestive organs were much smaller than those in wild-type (WT) embryos (Fig. 1b and Supplementary Fig. 1c). WISH of *insulin* and cross-section showed that the development of endocrine-pancreas was not obviously affected in *wdr5*^*−/−*^ mutant (Fig. 1b and Supplementary Fig. 1e).

WISH showed that *wdr5* mRNA was detected at one-cell stage and western blot showed that Wdr5 protein was detected as early as 3.5 hpf before the expression of zygotic genes (Fig. 1c and Supplementary Fig. 1a), demonstrating *wdr5* is a maternal gene. *wdr5* ubiquitously expressed at early embryogenesis and enriched in digestive organs from 2 dpf (Fig. 1c and Supplementary Fig. 1b). To investigate the functions of *wdr5* in digestive organogenesis, we compared transcriptomes between WT and *wdr5*^*−/−*^ mutant embryos at 3 dpf by RNA-seq (Supplementary Fig. 2a, b). Total 4017 genes down-regulated and 2707 genes up-regulated were identified in *wdr5*^*−/−*^ mutant (|log_2_FoldChange | ≥ 1, *P*_*adj*_ < 0.05). KEGG pathway analysis of the down-regulated genes showed that most of top 20 items were associated with different metabolic processes such as metabolic pathways, purine metabolism, steroid hormone biosynthesis, drug metabolism, metabolism of xenobiotics by cytochrome P450, pentose and glucuronate interconversions, ascorbate and aldarate metabolism, porphyrin metabolism, biosynthesis of unsaturated fatty acids, fatty acid elongation, primary bile acid biosynthesis and linoleic acid metabolism (Supplementary Fig. 2c). The other main pathways in the top 20 down-regulated items were associated with neuroactive ligand-receptor interaction, retinol metabolism, phototransduction, glycosphingolipid biosynthesis-globo and isoglobo series (Supplementary Fig. 2c). These down-regulated pathways were reflective of the fact that the *wdr5*^−/−^ mutant was severely defective in the development of digestive organs, eyes and head. Among the up-regulated genes, most of top 12 items were involved in RNA, protein biosynthesis processes. Interestingly, apoptosis and cell cycle pathways were also up-regulated in the *wdr5*^*−/−*^ mutant (Supplementary Fig. 2d). The results indicated that smaller digestive organs of the *wdr5*^*−/−*^ mutant might be caused by cell death rather than cell cycle arrest.

We searched for 508 digestive organ differentiation genes expressed in liver, intestine or pancreas and 120 genes related to regulation of digestive organogenesis including transcription factors, activators, signals and receptors (Supplementary Tables S5 and 6). Among the digestive organ differentiation genes, total 239 genes down-regulated and only 30 genes up-regulated were identified (Fig. 1d), whereas among the digestive organogenesis regulators, only 1 gene down-regulated and up to 30 genes up-regulated were identified in *wdr5*^−*/−*^ mutant (Fig. 1e). The 10 digestive organ differentiation marker genes, that were undetectable by WISH in *wdr5*^*−/−*^ mutant, were among the list of the down-regulation genes, demonstrating the reliability of the obtained data. *cdx1b*, a transcription factor responsible for intestine development, was the only down-regulated regulator. WISH showed that the expression of *cdx1b* was detected in the intestine of *wdr5*^*−/−*^ mutant at 2 dpf and 3 dpf (Fig. 1f). One of the reasons for the down-regulated expression of *cdx1b* might be due to smaller digestive organs in the mutant embryos. The expression patterns of other important transcription factors related to regulation of digestive organogenesis such as: *foxa3* and *gata6* (pan-regulators), *prox1* (liver), *hnf4α* (liver and intestine), *pdx1* (pancreas and intestine), *hhex* (liver and hepatopancreatic duct) in the *wdr5*^−*/−*^ mutant, were more similar to those in WT at early developmental stages (28 hpf or 2 dpf) and became smaller at late developmental stage (3 dpf) (Fig. 1f and Supplementary Fig. 2e).

All these data demonstrated that the specification of digestive organs including liver, exocrine-pancreas and intestine was relatively normal and their differentiation was impaired in *wdr5*^−/−^ mutant, and the undifferentiated state in the endodermal organs of *wdr5*^*−/−*^ mutant embryos was not due to the insufficient expression of these digestive organogenesis regulators.

### Wdr5-mediated H3K4me3 is required for the expression of differentiation genes in digestive organs

**Fig. 2:**
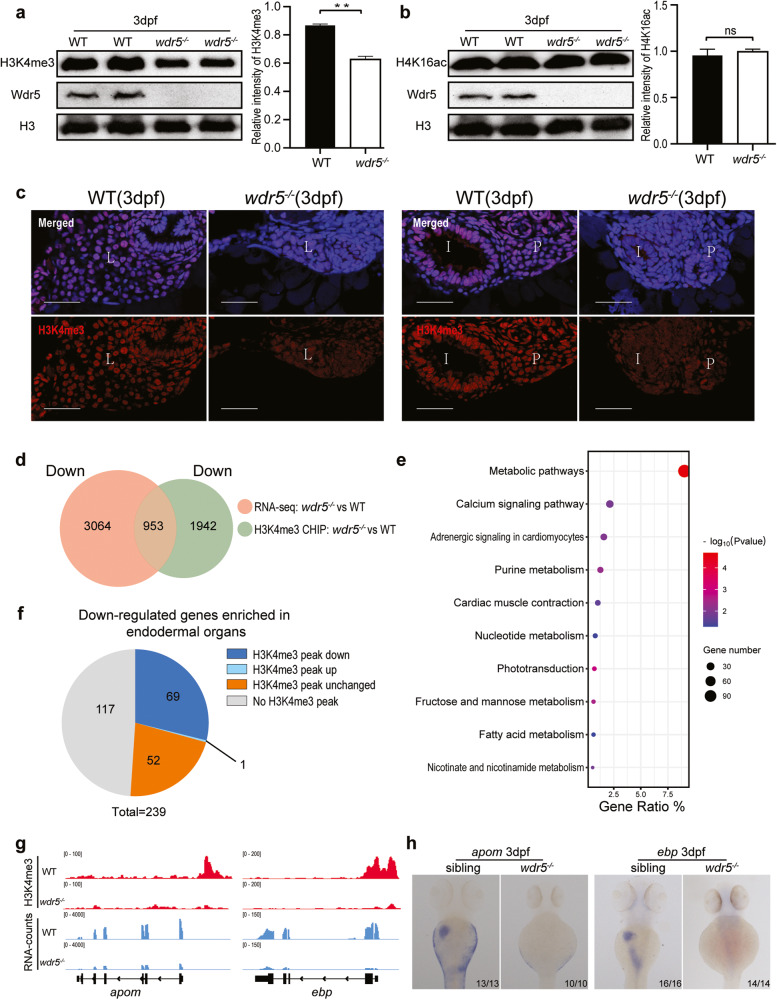
Wdr5 promotes the differentiation of digestive organs through H3K4me3, but not H4K16ac. **a** Western blot of H3K4me3 in WT and *wdr5*^*−/−*^ mutant embryos at 3 dpf. Relative intensity of H3K4me3 was normalized with H3. **b** Western blot of H4K16ac in WT and *wdr5*^*−/−*^ mutant embryos at 3 dpf. Relative intensity of H4K16ac was normalized with H3. **c** Cryosections of WT and *wdr5*^−*/−*^ mutant embryos at 3 dpf were immunostained by anti-H3K4me3 (in red). The nuclear was stained with DAPI (in blue). L: liver; I: intestine; P: pancreas. Scale bar: 40 μm. **d** Venn diagram showing 953 overlapping downregulated genes between RNA-seq (*wdr5*^*−/−*^ mutant vs WT, log_2_FoldChange ≤ −1, *P*_*adj*_ < 0.05) and H3K4me3 ChIP-seq (*wdr5*^*−/−*^ mutant vs WT, log_2_FoldChange ≤ −0.58, *P*_*adj*_ < 0.05). **e** KEGG analysis of overlapping downregulated genes between RNA-seq and H3K4me3 ChIP-seq in **d**. **f** Distribution of H3K4me3 peak changes (*wdr5*^*−/−*^ mutant vs WT, |log_2_FoldChange | ≥ 0.58, *P*_*adj*_ < 0.05) in 239 downregulated genes (*wdr5*^*−/−*^ mutant vs WT, log_2_FoldChange ≤ −1, *P*_*adj*_ < 0.05) enriched in digestive organs such as liver, intestine and exocrine pancreas. **g** Graphs showing H3K4me3 and RNA peaks at *apom* and *ebp* loci in WT and *wdr5*^*−/−*^ mutant embryos at 3 dpf. **h** WISH of *apom* and *ebp* in WT and *wdr5*^*−/*−^ mutant at 3 dpf. Each experiment was repeated for three times with similar results and a representative was showed here. *n* indicates the number of zebrafish embryos in each group. Data are mean ± S.D. Two-tailed *t-*test was applied for each individual comparison (**p < 0.01; n.s no significance).

Wdr5 has two main functions to facilitate H3K4me3 and H4K16ac modifications. Western blot showed that the total modification level of H3K4me3, but not H4K16ac, significantly decreased in *wdr5*^*−/−*^ mutant embryos at 3 dpf (Fig. 2a, b). Immunostaining confirmed that the signal intensity of H3K4me3 obviously decreased in liver, intestine and pancreas of *wdr5*^*−/−*^ mutant embryos at 3 dpf, while the signal intensity of H4K16ac did not (Fig. 2c and Supplementary Fig. 3a, b). The results indicated that Wdr5 regulates digestive organ differentiation through H3K4me3. Therefore, we performed ChIP-seq to compare the distribution of H3K4me3 on chromatins in WT and *wdr5*^*−/−*^ mutant embryos at 3 dpf (Supplementary Fig. 4a). Normalized reads distribution profiles showed that H3K4me3 was enriched around the transcription start sites (TSS) in WT and *wdr5*^−*/−*^ mutant embryos (Supplementary Fig. 4b). H3K4me3 peaks in 2895 genes significantly decreased and H3K4me3 peaks in 3356 genes significantly increased in *wdr5*^−*/−*^ mutant (|log_2_FoldChange | ≥ 0.58, *P*_*adj*_ < 0.05) (Fig. 2d and Supplementary Fig. 4c). Among 3356 genes with increased H3K4me3 peaks in *wdr5*^*−/−*^ mutant, 827 genes were also identified to be transcriptionally up-regulated by RNA-seq. KEGG pathway analysis of these shared up-regulated genes showed that most of top 12 items were associated with RNA, protein biosynthesis and stability. p53 signaling pathway and apoptosis were also enriched in the up-regulated genes (Supplementary Fig. 4c, d). The results suggested that the activation of p53 signaling might lead to apoptosis in *wdr5*^*−/−*^ mutant embryos.

Among 2895 genes with decreased H3K4me3 peaks in *wdr5*^*−/−*^ mutant, 953 genes were overlapped with down-regulated genes in RNA-seq (Fig. 2d). KEGG pathway analysis of these shared down-regulated genes showed that most of top 10 items were associated with different metabolic processes and phototransduction (Fig. 2e). Among 239 down-regulated endodermal differentiation genes in RNA-seq, H3K4me3 peaks were decreased in 69 genes (for instance, *apom* and *ebp*), not changed in 52 genes (*sdr16c5b*, *tprg1* etc), not detected in 117 genes (*fabp10a, fabp2, prss59.1, gc*, etc), and increased only in one gene (Fig. 2d, f, g and Supplementary Fig. 4g, h). The results suggested that the decrease of H3K4me3 level in the endodermal organs of *wdr5*^*−/−*^ mutant embryos (Fig. 2a, c) was correlated to the expression downregulation of many endodermal differentiation genes.

Among 120 digestive organogenesis regulators, H3K4me3 peaks were not changed in 65 genes, increased in 30 genes, not detected in 23 genes, decreased only in 2 genes (Supplementary Fig. 4e). H3K4me3 peaks were not detected in the only one downregulated endodermal transcriptional factor *cdx1* gene (Supplementary Fig. 4f). The data demonstrated that Wdr5-mediated H3K4me3 promotes endodermal organ differentiation not by up-regulating the expression of these digestive organogenesis regulators.

### Wdr5-mediated H3K4me3 is required for digestive organ differentiation

**Fig. 3:**
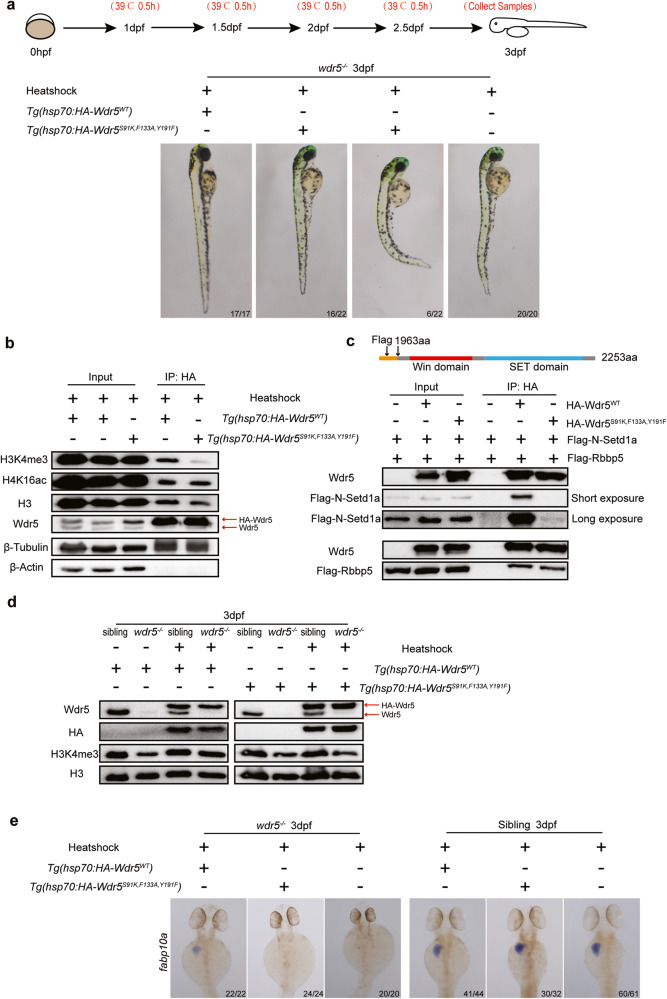
Wdr5-mediated H3K4me3 is required for digestive organ differentiation. **a** Two transgenic lines of *Tg(hsp70:HA-Wdr5*^*WT*^*)* and *Tg(hsp70:HA-Wdr5*^*S91K,F133A,Y191F*^*)* were treated with heat shock. Two transgenes of *Tg(hsp70:HA-Wdr5*^*WT*^*)* and *Tg(hsp70:HA-Wdr5*^*S91K,F133A,Y191F*^*)* were generated in *wdr5*^*+/*−^ background. The upper diagram showing how the heat shock treatment was performed. The embryos crossed from each transgenic line were treated with four times of heat shock (39 °C for half an hour) at 1, 1.5, 2, and 2.5 dpf, respectively. The treated embryos were sampled at 3 dpf. Representative images were taken before the genotyping. **b** Co-IP analysis of the interaction between HA-Wdr5 and H3, H3K4me3 or H4K16ac in two transgenetic lines: *Tg(hsp70:HA-Wdr5*^*WT*^*)* and *Tg(hsp70:HA-Wdr5*^*S91K,F133A,Y191F*^*)* under heatshock condition. HA beads were used for immunoprecipitation. β-Actin and β-Tubulin were used as the interaction negative and positive controls, respectively. More than 300 embryos of 1 dpf from each line were treated with heat shocks as described in Fig. 3a. **c** Co-IP of the interaction between Flag-N-Setd1a or Flag-Rbbp5 with the HA-Wdr5^WT^ or HA-Wdr5^S91K,F133A,Y191F^ in 293 T cells. The upper diagram showing Flag-tagged N terminal of zebrafish Setd1a protein used in Co-IP. Different plasmids were transfected or co-transfected into 293 T cells as indicated. HA beads were used for immunoprecipitation. A zebrafish Wdr5 antibody was used to detect HA-Wdr5^WT^ or HA-Wdr5^S91K,F133A,Y191F^. Flag antibody was used to detect Flag-Rbbp5 and Flag-N-Setd1a. **d** Western blots detected by anti-Wdr5, anti-HA, anti-H3K4me3 or anti-H3 antibody in different samples as indicated. Two transgenic lines *Tg(hsp70:HA-Wdr5*^*WT*^*)* and *Tg(hsp70:HA-Wdr5*^*S91K,F133A,Y191F*^*)* were generated in *wdr5*^*+/*−^ background. More than 200 embryos of 1 dpf from each line were treated with heat shocks as described in Fig. 3a. Treated embryos were genotyped before protein extraction. At least 30 embryos in each group were pooled together for protein extraction. **e** WISH of *fabp10a* in transgenic *wdr5*^*−/−*^ and sibling embryos treated with heat shocks as described in Fig. 3a. Each experiment was repeated for three times with similar results and a representative was showed here. *n* indicates the number of zebrafish embryos in each group.

Previous studies have demonstrated that Ser91, Phe133, and Tyr191 are key residues of human WDR5 to directly interact with the N-terminal of Histone 3 (H3) [29, 30]. The protein sequence alignment showed zebrafish Wdr5 shares a high similarity with human or mouse WDR5, and especially the three residues of Ser91, Phe133, and Tyr191 are also conserved among the three species (Supplementary Fig. 5a). To evaluate whether the interaction between Wdr5 and H3 plays an essential role in the differentiation of endodermal organs, we generated two transgenetic lines: *Tg(hsp70:HA-Wdr5*^*WT*^*)* and *Tg(hsp70:HA-Wdr5*^*S91K,F133A,Y191F*^*)* (Fig. 3a). Western blot showed that HA-Wdr5^WT^ or HA-Wdr5^S91K,F133A,Y191F^ was induced in corresponding transgenetic lines by heatshock treatments (Fig. 3d). Co-immunoprecipitation (Co-IP) showed that the mutations in three residues of zebrafish Wdr5 did not affect the complex formation with H3 and H4K16ac, but obviously impaired the H3K4me3 (Fig. 3b). The induction of HA-Wdr5^WT^ obviously increased the H3K4me3 level in *wdr5*^*−/−*^ embryos at 3 dpf, but not in *wdr5*^*+/−*^ embryos. However, the induction of HA-Wdr5^S91K,F133A,Y191F^ did not changed the H3K4me3 level in either *wdr5*^*−/−*^ or *wdr5*^*+/−*^ embryos at 3 dpf (Fig. 3d), suggesting that the three residues are also important for Wdr5 to facilitate H3K4me3 in zebrafish.

Studies from human cell lines have demonstrated that WDR5 is an adaptor protein in the COMPASS complex and can directly interact with N-terminal of SETD1A or RBBP5 [31, 32]. N terminal of zebrafish Setd1a shared a high similarity with human SETD1A (Supplementary Fig. 5b, c). To investigate if mutations in the three residues of Wdr5^S91K,F133A,Y191F^ interrupt the interaction between Wdr5 and Setd1a or Rbbp5, we co-transfected zebrafish HA-Wdr5 and Flag-N-Setd1a or Flag-Rbbp5 into 293 T cells, Co-IP showed that both HA-Wdr5^WT^ and HA-Wdr5^S91K,F133A,Y191F^ were able to interact with Rbbp5 (Fig. 3c). However, only HA-Wdr5^WT^, but not HA-Wdr5^S91K,F133A,Y191^ interacted with N-Setd1a (Fig. 3c). The results suggest that these three residues are important for Wdr5 to form complex with Setd1a, but not with Rbbp5. Furthermore, the abnormal phenotypes of *wdr5*^*−/−*^ embryos at 3 dpf including curved body, small eyes, the expression of digestive organ differentiation genes: *apom, ebp, fabp10a, fabp2, prss59.1* were almost completely rescued by the induction of HA-Wdr5^WT^, but not by the induction of HA-Wdr5^S91K,F133A,Y191F^ (Fig. 3a, e and Supplementary Fig. 5d).

Taken together, these data demonstrate that the interaction between Wdr5 and Setd1a is required for Wdr5 to mediate H3K4me3, which plays an essential role in zebrafish endodermal organ differentiation.

### Overactivation of Wnt/β-Catenin signal is responsible for the increase of cell proliferation in *wdr5*^−*/−*^ mutant digestive organs

**Fig. 4:**
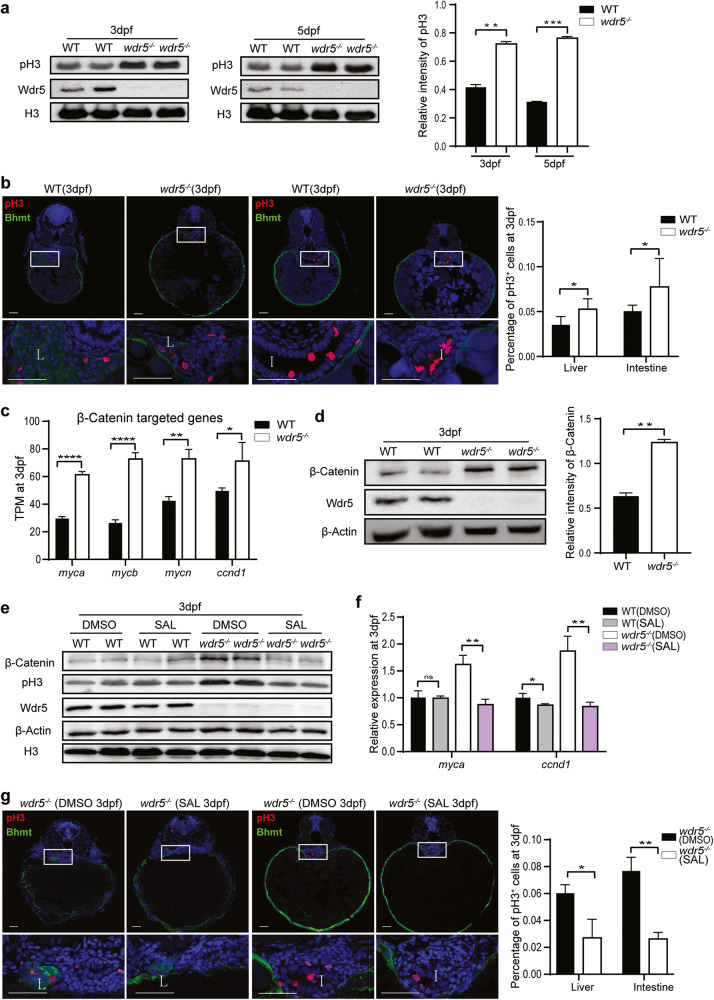
Overactivation of Wnt/β-Catenin signal is responsible for the increase of cell proliferation in *wdr5*^*−/*−^ mutant digestive organs. **a** Western blots of pH3, Wdr5, β-Actin, H3 in WT and *wdr5*^*−/−*^ mutant embryos at 3 and 5 dpf. Relative intensity of pH3 was normalized with H3. **b** Cryosections of WT and *wdr5*^*−/−*^ mutant embryos at 3 and 5 dpf were immunostained by anti-pH3 (in red) anti-Bhmt (a liver specific marker in green). The nuclear was stained with DAPI (in blue). L: liver; I: intestine. Framed area was magnified in bottom panel. Scale bar: 40 μm. The percentage of pH3 positive cells in each sample was calculated as the number of pH3 positive cells divided by total cell number in different organs from continuous cryosections. Also see Supplementary Fig. 6b. **c** Transcript TPM of β-Catenin targeted genes (*myca*, *mycb*, *mycn* and *ccnd1*) in RNA-seq from WT and *wdr5*^*−/−*^ mutant embryos at 3 dpf. **d** Western blots of β-Catenin, Wdr5, β-Actin, in WT and *wdr5*^*−/−*^ mutant embryos at 3 dpf. Relative intensity of β-Catenin was normalized with β-Actin. **e** Western blots of β-Catenin, pH3, Wdr5, β-Actin and H3 in WT and *wdr5*^*−/*−^ mutant embryos with different treatment as indicated. The *wdr5*^*−/−*^ mutant and WT embryos at 2.3 or 4 dpf were treated with SAL or DMSO. The protein was extracted from treated embryos at 3 and 5 dpf. Also see Supplementary Fig. 6d, e. **f** Relative expression level of *myca* and *ccnd1* in WT and *wdr5*^*−/−*^ mutant embryos at 3 dpf with SAL or DMSO. The treatment was described in Fig. 4e. **g** Cryosections of WT and *wdr5*^*−/−*^ mutant embryos at 3 dpf treated with SAL or DMSO were immunostained by anti-pH3 (in red) and anti-Bhmt (in green). The nuclear was stained with DAPI (in blue). L: liver; I: intestine. Framed area was magnified in bottom panel. Scale bar: 40 μm. Statistical analysis on the percentage of pH3 positive cells in the liver or intestine of *wdr5*^*−/−*^ embryos at 3 dpf treated with SAL or DMSO was showed in the right panel. The treatment was described in Fig. 4e. Each experiment was repeated for three times with similar results and a representative was showed here. *n* indicates the number of zebrafish embryos in each group. Data are mean±S.D. Two-tailed *t-*test was applied for each individual comparison (*p < 0.05; **p < 0.01; ***p < 0.001; ****p < 0.0001; n.s no significance).

Transcriptome analysis revealed that cell cycle was among the up-regulated items in the *wdr5*^*−/−*^ mutant embryos (Supplementary Fig. 2d). Among 6724 differentiated expression genes (DEGs) between WT and *wdr5*^*−/−*^ embryos, 123 cell cycle related genes were upregulated and only 46 cell cycle related genes were downregulated (Supplementary Fig. 6a). Western blot showed that total phospho-Histone3 (pH3, a mitosis marker) level significantly increased in the *wdr5*^−*/−*^ mutant embryos at 3 and 5 dpf (Fig. 4a). Immunostaining displayed that the percentages of pH3 positive cells also significantly increased in the liver and intestine of *wdr5*^−*/−*^ mutant embryos at 3 and 5 dpf (Fig. 4b and Supplementary Fig. 6b, c). The results demonstrated that cell proliferation was increased in the digestive organs of *wdr5*^*−/−*^ mutant embryos. In searching for the reasons of the increased proliferation, we found that the expression of β-Catenin target genes (such as: *myca*, *mycb*, *mycn* and *ccnd1* highly correlated to cell proliferation) was significantly up-regulated in *wdr5*^*−/−*^ mutant embryos from transcriptome analysis (Fig. 4c). Western blot showed that β-Catenin protein was increased in *wdr5*^*−/−*^ mutant embryos at 3 dpf (Fig. 4d). Previous studies have demonstrated that the Wnt /β-Catenin signal promotes hepatoblast and intestinal stem cell proliferation [33–35]. To investigate if the over activation of β-Catenin plays a role for the increased proliferation in *wdr5*^*−/−*^ mutant endodermal organs, we treated *wdr5*^*−/−*^ mutant embryos with Salinomycin sodium salt (SAL), a Wnt signal inhibitor (inhibiting phosphorylation of Lrp6-Wnt receptor) [36, 37]. The results showed that the treatment of SAL not only decreased the accumulation of β-Catenin and pH3, and the expression of β-Catenin-target genes (*myca* and *ccnd1*) in *wdr5*^*−/−*^ mutant embryos down to the levels of those in WT embryos, but also significantly reduced the percentage of pH3 positive cells in the liver and intestine of *wdr5*^*−/−*^ mutant embryos at 3 and 5 dpf, whereas the treatment of SAL did not have obvious effects on the accumulation of β-Catenin and pH3 in WT embryos at 3 and 5 dpf, suggesting that the over activation of β-Catenin was responsible for the increased proliferation in *wdr5*^*−/−*^ mutant endodermal organs (Fig. 4e–g and Supplementary Fig. 6d, e).

### Wdr5-mediated H3K4me3 upregulates *apc* expression to inhibit Wnt/β-Catenin signal, resulting cell cycle termination in differentiated cells of digestive organs

**Fig. 5:**
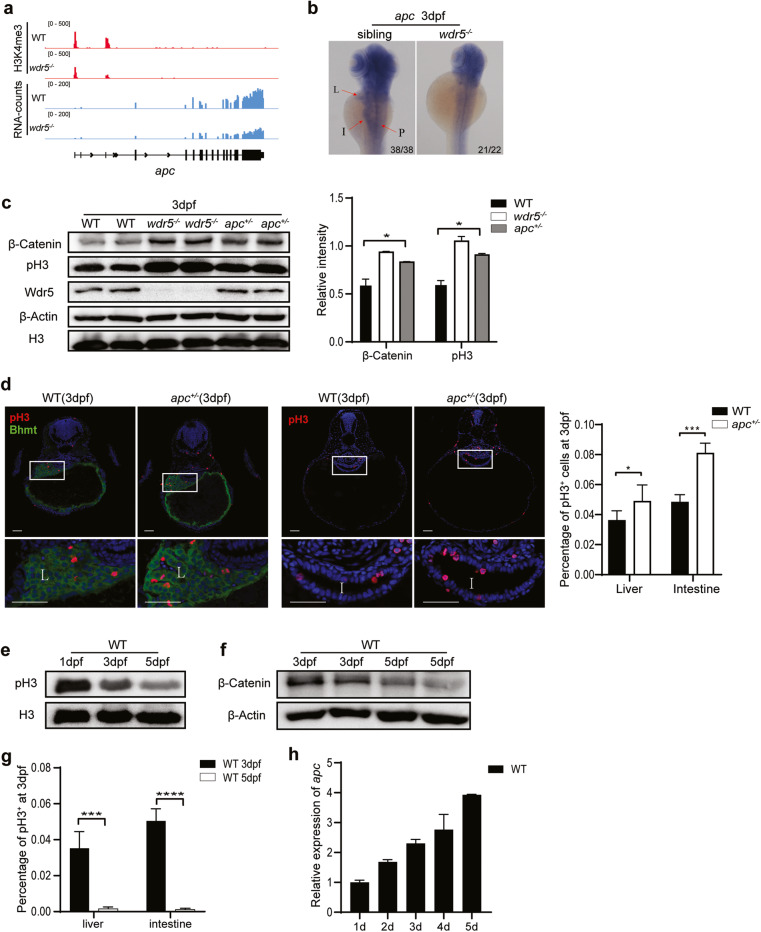
Wdr5-mediated H3K4me3 upregulates *apc* expression to inhibit Wnt/β-Catenin signal. **a** Graphs showing H3K4me3 and RNA peaks at *apc* locus in WT and *wdr5*^*−/−*^ mutant embryos at 3 dpf. **b** WISH of *apc* in WT and *wdr5*^−*/−*^ mutant at 3 dpf. L: liver; I: intestine; P: pancreas. **c** Western blots of β-Catenin, pH3, Wdr5, β-Actin and H3 in WT, *wdr5*^−*/−*^ and *apc*^*+/−*^ embryos at 3 dpf. Relative intensities of β-Catenin and pH3 in WT, *wdr5*^*−/−*^ and *apc*^*+/−*^ from the western blots were normalized with H3 was showed in right panel. **d** Cryosections of WT and *apc*^*+/−*^ embryos at 3 dpf were immunostained by anti-pH3 (in red) and anti-Bhmt (in green). The nuclear was stained with DAPI (in blue). L: liver; I: intestine. Framed area was magnified in bottom panel. Scale bar: 40 μm. Statistical analysis on the percentages of pH3 positive cells in the liver or intestine between WT and *apc*^*+/*−^ embryos at 3 dpf was showed in the right panel. **e** Western blots of pH3 and H3 in WT embryos at 1 dpf, 3 dpf and 5 dpf. **f** Western blots of β-Catenin and β-Actin in WT embryos at 3 dpf and 5 dpf. **g** Statistical analysis on the percentages of pH3 positive cells in liver and intestine of WT between 3 dpf and 5 dpf. **h** Relative expression of *apc* in WT embryos from 1–5 dpf. Each experiment was repeated for three times with similar results and a representative was showed here. *n* indicates the number of zebrafish embryos in each group. Data are mean±S.D. Two-tailed *t-*test was applied for each individual comparison (*p < 0.05; ***p < 0.001, ****p < 0.0001, n.s no significance).

To find out why β-Catenin protein increased in *wdr5*^*−/−*^ mutant embryos, we examined the expression of upstream regulators of β-Catenin including *ctnnb1* encoding β-Catenin (Supplementary Fig. 7a), wnt ligands and members of β-Catenin degradation complex from transcriptome analysis. Interestingly, we found that the expression of *Adenomatous Polyposis Coli* (*apc*), a member of β-Catenin degradation complex, decreased up to 35%, while the H3K4me3 peaks in *apc* locus were also decreased about 33% in *wdr5*^*−/−*^ mutant embryos (Fig. 5a). WISH showed that the expression of *apc* enriched in head and endodermal organs of WT embryos at 3 dpf (Fig. 5b). However, no expression enrichment was observed in endodermal organs of *wdr5*^*−/−*^ mutant embryos at 3 dpf (Fig. 5b). The results suggested that downregulation of *apc* expression was due to the decrease of Wdr5-mediated H3K4me3 in endodermal organs. A previous study showed that a zebrafish *apc*^+/−^ heterozygous mutant embryos developed enlarged livers through increased proliferation [38]. To evaluate whether this *apc* haploid insufficiency also results increased proliferation in other digestive organs, we used another *apc* mutant allele (Supplementary Fig. 7c). The homozygous mutation of *apc*^*−/−*^ led to early embryo lethality. Interestingly, similar to the *wdr5*^*−/−*^ mutant embryos, the levels of total β-Catenin and pH3 were also increased in *apc*^*+/*−^ mutant embryos at 3 dpf (Fig. 5c), whereas the expression of *apc* in both of *apc*^*+/*−^ and *wdr5*^*−/−*^ embryos was significantly lower than that in WT embryos at 3 dpf (Supplementary Fig. 7d). The percentages of pH3 positive cells were significantly increased in liver and intestine of *apc*^*+/−*^ mutant embryos at 3 dpf (Fig. 5d).

To evaluate if Wdr5 promotes *apc* expression to terminate digestive organ proliferation through H3K4me3, we performed western blot to examine the levels of β-Catenin protein and pH3, and quantitative Real-Time PCR (qRT-PCR) to analyze the transcripts of *apc* and *β-catenin* in two transgenetic lines: *Tg(hsp70:HA-Wdr5*^*WT*^*)* and *Tg(hsp70:HA-Wdr5*^*S91K,F133A,Y191F*^*)* under *wdr5*^*−/−*^ genetic background. The qRT-PCR showed that the induction of HA-Wdr5^WT^, but not HA-Wdr5^S91K,F133A,Y191F^, up-regulated the expression of *apc*, while both transgenes had a little effect on the expression of *β-catenin* (Supplementary Fig. 7e). Western blot displayed that only HA-Wdr5^WT^ down-regulated the levels of β-Catenin protein and pH3 (Supplementary Fig. 7f). Taken together, the data demonstrated that Wdr5-mediated H3K4me3 repressed cell proliferation through promoting the expression of *apc* to degrade β-Catenin protein.

In addition, we observed that the expression of *apc* was gradually increased from 1 to 5 dpf (Fig. 5h), and the enrichment of *apc* expression in endodermal organs was also gradually increased from 1 to 5 dpf in WT embryos (Supplementary Fig. 7b), while the levels of total β-Catenin and pH3 were decreased from 3 to 5 dpf in WT embryos (Fig. 5e, f). The percentage of pH positive cells in either liver or intestine of WT embryos was greatly reduced from 3 to 5 dpf (Fig. 5g). The results indicated that on the one hand, Wdr5-mediated H3K4me3 promoted the expression of digestive organ differentiation genes; on the other hand, Wdr5-mediated H3K4me3 decreased the cell proliferation in differentiated cells by promoting *apc* expression.

### The increase of apoptotic activity in *wdr5*^*−/−*^ mutant is largely dependent on the activation of p53

**Fig. 6:**
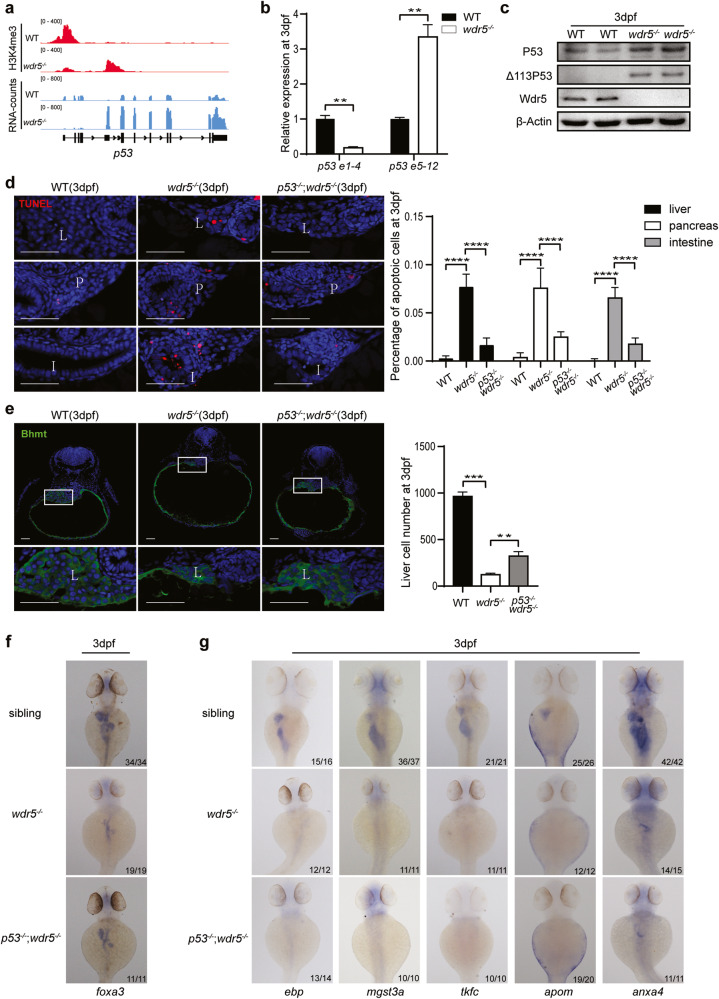
The increase of apoptotic activity in *wdr5*^*−/−*^ mutant is largely dependent on the activation of p53. **a** Graphs showing H3K4me3 and RNA peaks at *p53* locus in WT and *wdr5*^*−/−*^ mutant embryos at 3 dpf. **b** The confirmation of the expression levels of full-length *p53* transcript (Exon 1-4, only in full-length *p53*) and *Δ113p53* transcript (Exon 5–12 in both full-length *p53* and *Δ113p53*) by qRT-PCR. **c** Western blots detected by anti-p53 (for both p53 50 kD and Δ113p53 35kD), anti-Wdr5, or anti-β-Actin antibody in WT and *wdr5*^−*/−*^ mutant embryos at 3 dpf. **d** Cryosections of WT, *wdr5*^*−/−*^ and *p53*^*−/−*^;*wdr5*^*−/*−^ embryos at 3 dpf were analyzed by TUNEL assay (in red). The nuclear was stained with DAPI (in blue). Statistical analysis on the percentages of apoptotic cells in the liver, pancreas or intestine between genotypes was showed in the right panel. L: liver; P: exocrine pancreas; I: intestine. **e** Cryosections of WT, *wdr5*^*−/−*^ and *p53*^*−/−*^;*wdr5*^*−/−*^ embryos at 3 dpf were immunostained by anti-Bhmt (in green). The nuclear was stained with DAPI (in blue). Framed area was magnified in bottom panel. Scale bar: 40 μm. Total cell number of liver was counted from the Bhmt-positive cells in continuous cryosections. Statistical analysis on the total cell numbers of liver between genotypes was showed in the right panel. **f** WISH of WT, *wdr5*^*−/−*^ and *p53*^*−/−*^;*wdr5*^*−/−*^ embryos at 3 dpf with *foxa3*. **g** WISH of WT, *wdr5*^*−/*−^ and *p53*^*−/−*^;*wdr5*^*−/−*^ embryos at 3 dpf with *ebp, mgst3a, tkfc, apom* and *anxa4* as indicated. Each experiment was repeated for three times with similar results and a representative was showed here. *n* indicates the number of zebrafish embryos in each group. Data are mean ± S.D. Two-tailed *t-*test was applied for each individual comparison (**p < 0.01; ***p < 0.001, ****p < 0.0001; n.s no significance).

Next question is why the digestive organs in *wdr5*^*−/−*^ mutant embryos are smaller, even though their cell proliferation rate is higher, than those in WT embryos. Our previous studies have demonstrated that Δ113p53 is an N-terminal truncated p53 isoform that lacks exon 1 to 4 [10]. It is a p53 target gene and transcribed from an alternative promoter in intron 4 [10]. Transcriptome analysis showed that the expression of exon 1-4 of *p53* gene was decreased and the expression of exon 5-12 of *p53* was increased in *wdr5*^*−/−*^ mutant embryos, which was confirmed by qRT-PCR (Fig. 6a, b). Furthermore, the accumulation of H3K4me3 was decreased around exon1 and increased in intron4 (Fig. 6a). The results demonstrated that the transcription of the full-length *p53* was decreased and the transcription of its target gene *Δ113p53* was increased. The expression of other p53 target genes (including *p21*, *mdm2*, *bax*, *gadd45ba*) was also increased in *wdr5*^*−/−*^ mutant embryos (Supplementary Fig. 8a). Western blot revealed that p53 protein obviously increased in *wdr5*^*−/−*^ mutant embryos at 3 dpf (Fig. 6c). These results were consistent with KEGG analysis, which showed that the p53 signal pathway in *wdr5*^*−/−*^ mutant embryos was among the items of upregulated genes in both RNA-seq and H3K4me3 ChIP-seq (Supplementary Figs. 2d and 4d). To investigate whether the activation of p53 played a role in small digestive organs of *wdr5*^*−/−*^ mutant embryos, we generated *p53*^−*/−*^;*wdr5*^−*/−*^ double mutant. The abnormal phenotypes in *wdr5*^*−/*−^ mutant (curved body, small eyes) were partially rescued in the *p53*^*−/−*^;*wdr5*^*−/−*^ mutant (Supplementary Fig. 8b). TUNEL assay showed that the percentages of apoptotic cells in liver, intestine and pancreas at 3 dpf were significantly increased in *wdr5*^*−/−*^ mutant, compared with those in WT embryos, whereas the apoptotic activities in the three digestive organs were significantly reduced in *p53*^*−/−*^;*wdr5*^*−/−*^ mutant, compared with *wdr5*^*−/−*^ mutant (Fig. 6d and Supplementary Fig. 8c). Furthermore, the total cell number of liver and the size of *foxa3* expression (a pan marker for liver, pancreas and intestine) at 3 dpf were partially rescued in *p53*^*−/−*^;*wdr5*^*−/−*^ mutant, compared with *wdr5*^−*/−*^ mutant, while western blot showed that β-Catenin and pH3 in *p53*^*−/−*^;*wdr5*^−*/−*^ mutant remained similar levels as those in *wdr5*^*−/−*^ mutant (Fig. 6e, f and Supplementary Fig. 8d, e). The results demonstrated that one of main reasons for the small digestive organs in *wdr5*^*−/−*^ mutant was the activation of apoptotic activity, which was triggered by the p53 protein accumulation.

Interestingly, the expression of digestive organ differentiation genes (such as *apom*, *ebp*, *mgst3a*, *tkfc* and *anxa4*), that were downregulated in *wdr5*^*−/*−^ mutant from both RNA-seq and H3K4me3 ChIP-seq (Fig. 2g and Supplementary Fig. 8f), was not restored in *p53*^*−/−*^;*wdr5*^*−/−*^ mutant (Fig. 6g). The results indicated that small undifferentiated digestive organs in *wdr5*^*−/−*^ mutant were caused by the apoptosis of undifferentiated cells, but not by the apoptosis of differentiated cells. The expression of the differentiation genes depended on both their specific transcription factors and the H3K4me3 modification on their transcriptional start site. If a cell under differentiated status was not able to differentiate, the cell would undergo apoptosis.

### Wdr5-mediated H3K4me3 promotes differentiated cell survival partially by upregulating the expression of *xiap-like* gene

**Fig. 7:**
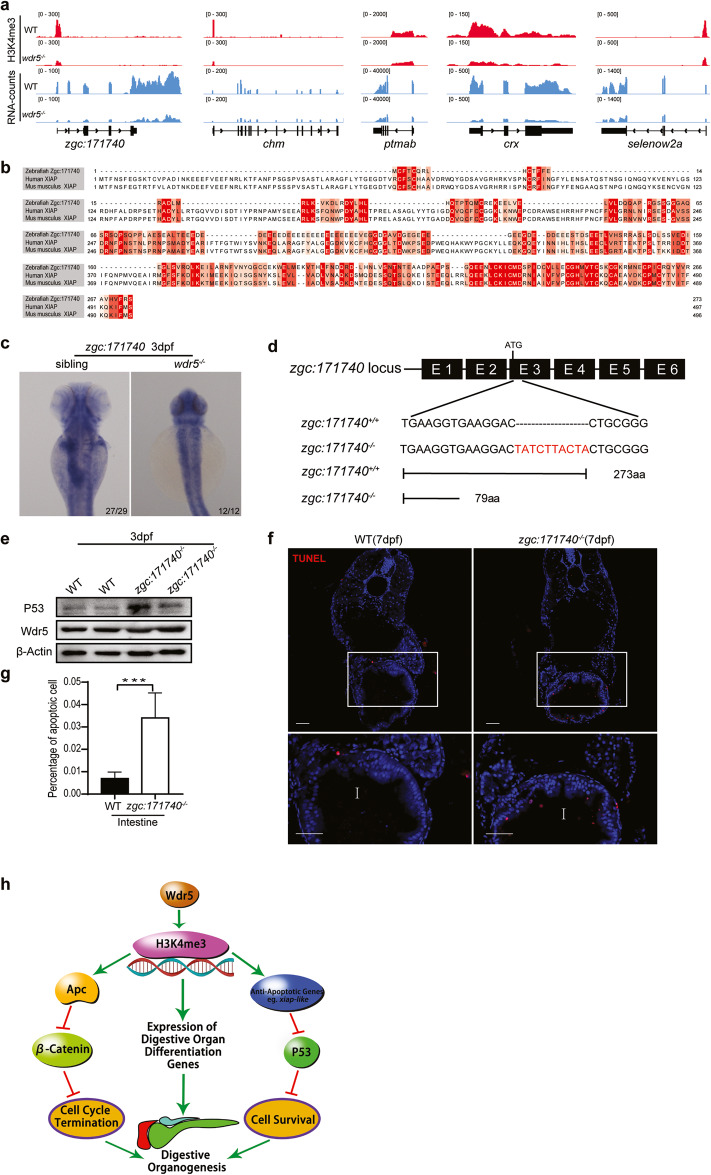
Wdr5-mediated H3K4me3 promotes differentiated cell survival partially by upregulating the expression of *xiap-like* gene. **a** Graphs showing H3K4me3 and RNA peaks at 5 anti-apoptotic gene loci (*zgc:171740, chm, ptmab, crx* and *selenow2a*) in WT and *wdr5*^−*/−*^ mutant embryos at 3 dpf. Both H3K4me3 and RNA peaks at these 5 gene loci were decreased in *wdr5*^*−/−*^ mutant embryos. **b** Amino acid sequence alignment between zebrafish Zgc:171740 (Danio rerio NP_001103196), human XIAP (Homo sapiens NP_001158) and Xiap (Mus musculus NP_001288568). **c** WISH of *zgc:171740* in WT and *wdr5*^*−/−*^ embryos at 3 dpf. **d** Diagram showing the gRNA targeting site and 10-bp insertion (in red) in the exon 3 of *zgc:171740* mutant, which results in a premature stop codon (PTC) at 79 aa. ATG: translation start codon. **e** Western blots of p53, Wdr5 and β-Actin in WT and *zgc:171740*^−*/−*^ mutant embryos at 3 dpf. **f**, **g** Cryosections of WT and *zgc:171740*^*−/−*^ mutant embryos at 7 dpf were analyzed by TUNEL assay (in red) and the nuclear was stained with DAPI (in blue) (**f**). Framed area was magnified in bottom panel. I: intestine. Scale bar: 40 μm. Statistical analysis on the percentages of apoptotic cells in the intestine between WT and *zgc:171740*^*−/−*^ mutant was in (**g**). **h** Model of the functions of Wdr5 in digestive organogenesis. Each experiment was repeated for three times with similar results and a representative was showed here. *n* indicates the number of zebrafish embryos in each group. Data are mean±S.D. Two-tailed *t-*test was applied for each individual comparison (***p < 0.001; n.s no significance).

The above results showed that the transcription of *p53* was decreased and its protein was increased in *wdr5*^*−/−*^ mutant, suggesting that p53 protein negative regulators might be downregulated in *wdr5*^*−/−*^ mutant. Therefore, we searched for downregulated anti-apoptotic genes in *wdr5*^*−/−*^ mutant from both RNA-seq and H3K4me3 ChIP-seq, and found that only 5 anti-apoptotic genes in this category including *choroideremia* (*chm*), *zgc:171740*, *prothymosin α type b* (*ptmab*), *cone-rod homeobox* (*crx*) and *selenoprotein W, 2a* (*selenow2a*) (Fig. 7a). Previous studies have demonstrated that *chm*, *ptmab*, *crx* and *selenow2a* promote cell survival during zebrafish development. However, their results also suggest that the survival role of these four genes is unlikely to be realized by regulation of the p53 protein activity [39–42]. Interestingly, protein alignment revealed that the Zgc:171740 protein shares 21.8% and 23.9% similarities with human and mouse X-linked inhibitor of apoptosis proteins (XIAP) respectively (Fig. 7b). A previous study has showed that XIAP directly interacts with p53 protein to downregulate p53 protein stability and mitochondrial localization [43]. WISH showed that the expression of *zgc:171740* was enriched in the liver, intestine and exocrine pancreas of WT embryos at 3 dpf, but disappeared in these digestive organs of *wdr5*^*−/−*^ mutant embryos (Fig. 7c). Next, we generated a zebrafish *zgc:171740* mutant (Fig. 7d). Western blot showed that the accumulation of p53 protein, but not Wdr5, obviously increased in *zgc:171740*^−/−^ mutant embryos at 3 dpf (Fig. 7e). TUNEL assay also revealed that the percentage of apoptotic cells was significantly increased in *zgc:171740* mutant intestine at 7 dpf (Fig. 7f, g). Although *zgc:171740*^−/−^ mutant fish developed relative normal at early stage, most of them (80–90%) died around 30 dpf. Thus, we named *zgc:171740* as *xiap-like* gene.

Taken together, the data demonstrated that Wdr5-mediated H3K4me3 inhibits p53 protein activity via upregulating the expression of anti-apoptotic genes, for example, partially through *xiap-like*, to promote digestive organ differentiated cell survival.

## Discussion

In summary, we have revealed a novel mechanism by which Wdr5 coordinately regulates digestive organogenesis such as intestine, liver and exocrine pancreas (Fig. 7h). Wdr5 facilitates its functions by mediating H3K4me3. H3K4me3 promotes the maturation of digestive organ differentiation in three directions: first, it promotes organ differentiation by upregulating the expression of differentiation genes; second, it inhibits Wnt/β-Catenin signal by upregulating the expression of *apc*, thus driving differentiated cells to terminate cell proliferation; third, it represses p53 protein activity by upregulating of anti-apoptotic gene expression (such as *xiap-like*), thus ensuring differentiated cell survival.

Knockout *Wdr5* mouse is heterozygous lethal at early embryonic stages before E8.5 [27]. Most of studies on the functions of Wdr5 in development were performed with embryonic stem cell culture [20, 21]. In early zebrafish embryonic development, H3K4me3 modification is co-occupied with H3K27me3 before zygotic genome activation (ZGA). Prior to ZGA, patterns of genes that initialize ZGA are converted to non-repressive states through discard H3K27me3 and retain H3K4me3 to activate gene expression [44]. However, due to lack of proper knockout of animal model, the functions of Wdr5-mediated H3K4me3 in organogenesis are rarely explored. Here, we have taken advantages of *wdr5* zebrafish knockout mutant, which develops relatively normal before organogenesis, as *wdr5* is a maternal gene. Our results show that *wdr5* promotes digestive cell differentiation and termination of differentiated cell proliferation.

Previous studies have demonstrated that H3K4me3 in promoter regions of differentiation marker genes is significantly enhanced during differentiation of pancreatic cells and hepatocytes from human Embryonic Stem Cells (hESCs) in vitro [45, 46]. A strong correlation has been also observed between expression of differentiation genes and presence of H3K4me3 on gene promoters during zebrafish intestinal development [47]. Here, we reveal that Wdr5 is required for H3K4me3 during digestive organogenesis, which plays an essential role in the expression of differentiation genes.

Abundant evidence has showed that the Wnt /β-Catenin signal plays an essential role in cell proliferation of endoderm-derived organs such as: intestine, liver and pancreas [48, 49]. APC is a well-known tumor suppressor and most commonly mutated in colorectal cancers, as well as many other types cancers like breast, pancreatic, liver and lung cancer [50]. Conditional knockout Apc in mouse results in the rapid nuclear relocalization of β-Catenin, failure to migrate and differentiate, and a “crypt progenitor-like” phenotype in intestine [51]. APC likely mediates tumor suppressions by negatively regulating the Wnt/β-catenin pathway [52]. Mutations in APC have been identified in early stages of cancer development making it a gatekeeper of tumor progression and therefore an ideal therapeutic target [52, 53]. However, it is unclear how the expression of *apc* in digestive organs is regulated. Here, we show that Wdr5-mediated H3K4me3 promotes *apc* expression to attenuate Wnt /β-Catenin signal to repress differentiated cell proliferation. The finding suggests that loss of H3K4me3 might also lead to tumorigenesis in these digestive organs through downregulation of *apc*.

Tumor repressor p53 is required to be tightly controlled during digestive organogenesis. Our previous studies have demonstrated that loss-of-function of *def* results in accumulation of p53 protein to lead to cell cycle arrest in digestive organs such as: liver, intestine and pancreas [10]. Def forms a complex with Capn3 to promote p53 protein degradation [54]. XIAP deficiency is a rare primary immunodeficiency and characterized by immune dysregulation and a broad spectrum of clinical manifestations, including haemophagocytic lymphohistiocytosis (HLH) and inflammatory bowel disease (IBD), etc [55]. XIAP is initially identified as a caspase-binding protein to be primarily involved in blocking apoptosis [56]. However, XIAP also prevents TNF-mediated, RIPK3-dependent cell death, by controlling RIPK1 ubiquitylation and preventing inflammatory cell death [57]. A recent study has shown that XIAP deficiency leads Paneth and dendritic cell disfuction by upregulating TNF-driven intestinal inflammation [58]. In this report, we find that zebrafish *xiap-like* expression is enriched in digestive organs to promote differentiated cell survival partially through inhibiting p53 protein activity, indicating that one of reasons for intestinal diseases caused by XIAP deficiency might be the activation of p53.

Therefore, our findings also provide mechanistic advances for understanding human diseases in these digestive organs.

## Materials and methods

### Zebrafish lines and maintenance

Zebrafish (Danio rerio) WT Tübingen strain was used in this study. All genetic mutants, except *apc*^*+/Δ8*^ and *p53*^*Δ10/Δ10*^ in the AB background, were generated in the Tübingen background unless otherwise mentioned. The animals were raised and maintained according to standard procedures described in ZFIN (http://www.zfin.org).

### Generation of transgenic fish

The coding sequence (cds) of *wdr5* was amplified from complementary DNA (cDNA) of WT embryos at 3 dpf. The miniTol2-*hsp70* fragment was Not I digesting and purification from miniTol2-*hsp70-cdx1b* plasmid [59]. To generate *HA-Wdr5*^*S91K,F133A,Y191F*^ mutant, different pairs of primers with different mutations such as S91K, F1331, Y191F, were designed to amplify respective regions of *wdr5* cds. For generating miniTol2-*hsp70:HA-Wdr5*^*WT*^ plasmid, different DNA fragments including the cds of *wdr5* was ligated to miniTol2- *hsp70* vector carrying SV40 terminator by one step of the homologous recombination method according to the manufacturer’s instructions (Vazyme). The construction of miniTol2-*hsp70:HA-Wdr5*^*S91K,F133A,Y191F*^ plasmid was similar to that of miniTol2-*hsp70:HA-Wdr5*^*WT*^ plasmid, in which the different mutated fragments of *wdr5* were used to replace the WT cds of *wdr5*. All constructs were confirmed by DNA sequencing. About 40 pg of each plasmid and 40 pg of Tol2 mRNA were co-injected into one-cell stage fertilized eggs to generate Tg F_0_ fish. Transgenic fish was screened by PCR with a pair of the transgene specific primers in F_1_ and were used thereafter. The information of all the primers was provided in the Supplementary Table S1.

### Generation of zebrafish genetic mutants

For the generation of *zgc:171740* and *p53* genetic mutants, the CRISPR/Cas9 technique was applied [60]. The zebrafish Cas9 expression plasmid pGH-T7-*zCas9* was kindly provided by Prof. Zhang Bo at College of Life Sciences, Peking University. The target sites of *zgc:171740*, *p53* and *apc* were provided in the Supplementary Table S1. The genomic region flanking gRNA target sites of each gene was amplified with a pair of gene specific primers (Supplementary Table S1). The amplified DNA fragments of the 2 genes were digested with different enzymes and subsequently confirmed by sequencing. The *apc* genetic mutant was purchased from China Zebrafish Resource Center. The information of all the primers was provided in the Supplementary Table S1.

### RNA isolation, reverse transcription and quantitative real-time PCR (qRT-PCR)

Total RNA was isolated using TRIZOL reagent (AidLab) according to the manufacturer’s protocol. For reverse transcription and qRT-PCR, RNA was digested with DNase I (NEB) and then purified with absolute alcohol and lithium chloride. Reverse transcription was carried with reverse transcriptase M-MLV (Invitrogen) according to the manufacturer’s protocol. qRT-PCR was performed in a CFX96TM Real-Time System (Bio-Rad) using a C1000 Thermal Cycler (Bio-Rad) with AceQ qRT-PCR SYBR Green Master Mix (Vazyme) according to the manufacturer’s instructions. Total RNA was normalized to the zebrafish *actb1* gene. The information of all the primers was provided in the Supplementary Table S1. Each qRT-PCR was repeated for three times with similar results and a representative was showed, More than 30 zebrafish embryos were used in each samples.

### Western blot

Total proteins were harvested from zebrafish embryos and extracted using standard SDS lysis buffer. Western blot was performed with following antibodies: anti-Wdr5, anti-β-Actin, anti-H3K4me3, anti-H3, anti-H4K16ac, anti-β-Catenin, anti-pH3, anti-p53. Relative intensity was quantified with ImageJ [61]. The information of all antibodies was provided in the Supplementary Table S7. Each Western blot was repeated for three times with similar results and a representative was showed, More than 30 zebrafish embryos were used in each samples.

### Whole mount in situ hybridization (WISH)

For WISH, zebrafish embryos were cultured in egg water with 0.004% PTU (Sigma) from 12 hpf. WISH was performed as previously described with following antisense probes labeled by digoxigenin (DIG): *fabp10a*, *gc*, *prss1*, *ela2l*, *fabp2*, *chia.1*, *wdr5*, *prox1a*, *cdx1b*, *foxa3*, *fads2*, *ctrb1*, *insulin*, *cdh17*, *slc6a19a.1*, *hnf4a*, *cdx1*, *hhex*, *gata6*, *apom*, *ebp*, *apc*, *zgc:171740* [10]. The information of all the primers to amplify the gene specific probes was provided in the Supplementary Table S1.

### Co-immunoprecipitation (Co-IP)

For Co-IP experiment in zebrafish, two transgenetic *Tg(hsp70:HA-Wdr5*^*WT*^*)* and *Tg(hsp70:HA-Wdr5*^*S91K,F133A,Y191F*^*)* were used. More than 300 zebrafish embryos from each line were treated with heatshock at 3 dpf. Zebrafish embryos were de-yolk by 2 ml Rnase-free niddle more than 15 times in PBS. The sediment were obtained at 12000 r.p.m. for 5 min at 4 °C, and then re-suspended in Co-IP lysis buffer on ice. Then suspended solution was homogenized at 60 s, 60 hz for 2 times and transferred into ice for 15 min, finally sonicated to break the nucleus and release the protein. The supernatant was obtained by centrifugation. HA-beads were used to capture HA-Wdr5 protein overnight at 4 °C with rotation.

For Co-IP experiment in 293 T cell line, 1 μg CMV-HA-Wdr5^WT^ (in pCS2+ vector) or 1 μg CMV-HA-Wdr5^S91K,F133A,Y191F^ was transfected or co-transfected with 1 μg CMV-Flag-Rbbp5 or 1 μg CMV-Flag-N-Setd1a into 293 T cells. The total amount of plasmid DNA transfected was 2 μg, any deficiency was topped up with pCS2+. Transfected cells were cultivated for 24 h at 37 °C followed by protein extraction using IP lysis buffer. HA beads were used for immunoprecipitation. For western blot analysis, Wdr5 antibody was used to detect HA-Wdr5^WT^ and HA-Wdr5^S91K,F133A,Y191F^. Flag antibody was used to detect Flag-Rbbp5 and Flag-N-Setd1a. Each Co-IP experiment was repeated for three times with similar results and a representative was showed.

### Immunostaining and histological methods

The cryosection immunostaining was performed as previously described [54]. The primary antibodies were anti-Wdr5, anti-H3K4me3, anti-H4K16ac, anti-pH3, anti-Bhmt and anti-β-Catenin. DAPI was used to stain nuclear. Images from different samples in each experiment were taken under the same voltage for respective laser channel by a confocal microscope (Olympus BX61WI). The percentage of pH3 positive cells in each sample was calculated as the number of pH3 positive cells divided by total cell number in different organs from continuous cryosections.

The information of all antibodies and the numbers of different cells from each sample were provided in the Supplementary Tables S2, 3 and 7, respectively.

Hematoxylin-Eosin (H&E) staining was performed as previously described [62] . Images were captured under an Olympus BX53 microscope with a camera from Qimaging MicroPublisher 5.0 RTV.

### TUNEL assay

The TUNEL assay was performed on freshly prepared cryosections using the in situ cell death detection kit, TMR red (Roche Diagnostics, 12156792910) according to the manufacturer’s instructions [6, 13, 14].

The information of different cells from each sample was provided in the Supplementary Table S4. More than 5 zebrafish embryos were used in each sample.

### Salinomycin sodium salt (SAL) treatment

Zebrafish embryos at 2.3 dpf were transferred into 6-well plate with 30 embryos per well. Unhatched embryos were manually dechorionated before SAL treatment to ensure effective drug penetration. The dechorionated embryos were treated with 1 μM SAL dissolved in egg water with 0.01% DMSO from 2.3 dpf to 3 dpf at 29 °C.

### RNA-seq analysis

Three independent RNA samples from WT and *wdr5*^*−/−*^ mutant embryos at 3 dpf were used for RNA-seq analysis. Isolation of mRNA, library construction, high throughput sequencing, data filtering, genome mapping (Danio_rerio.GRCz11) [63] and differential expression genes (DEGs) analysis (|log_2_FoldChange | ≥ 1 and *P*_*adj*_ < 0.05) were performed by Annoroad company (Beijing, China). KEGG analysis was further performed using DAVID Bioinformatics resources (https://david.ncifcrf.gov).

Genes related to endodermal organ differentiation and regulators of endodermal organ development were searched from the database of ZFIN (http://www.zfin.org) and published literatures.

### Chromatin immunoprecipitation followed by sequencing (ChIP-seq) analysis

Two independent samples from WT and *wdr5*^*−/−*^ mutant embryos at 3 dpf were used for ChIP-seq analysis. ChIP-seq and basic analysis including peak calling, peak annotation and differential analysis (|log_2_FoldChange | ≥ 0.58 and *P*_*adj*_ < 0.05) were performed by ActiveMotif Biotechnology company (Shanghai, China). KEGG analysis was further performed using DAVID Bioinformatics resources (https://david.ncifcrf.gov). Up-or down-regulated genes in both RNA-seq and ChIP-seq were found as |log_2_FoldChange | ≥ 1 in RNA-seq and |log_2_FoldChange | ≥ 0.58 in ChIP-seq with *P*_*adj*_ < 0.05 in both analysis (too few results with |log_2_FoldChange | ≥ 1 in ChIP-seq).

### Sampling of experiments

More than 5 pairs of mutant or WT fish were used to set one specific cross. For a cross between homozygous parents, more than 30 embryos were used for gene expression analysis in each treatment. For a cross between heterozygous parents, more than 50 embryos were used for each treatment and subjected for genotyping. Each experiment was repeated at least 3 times. A representative one was shown in the figures.

### Statistical analysis

Significance of differences between means was analyzed using two-sided *t*-test. Sample sizes were indicated in the figures or figure legends. Plotted mean was calculated by GraphPad software. Data were shown as mean ± SD. *P* value below 0.05 marked as *, *P* value below 0.01 marked as **, *P* value below 0.001 marked as ***, and *P* value below 0.0001 marked as ****; ns means no significant difference.

## Supplementary information

Supplementary Fig 1

Supplementary Fig 2

Supplementary Fig 3

Supplementary Fig 4

Supplementary Fig 5

Supplementary Fig 6

Supplementary Fig 7

Supplementary Fig 8

supplementary figure legends

author contribution

Original Data File

## Data Availability

All relevant data are available from the authors and/or included in the manuscript or Supplementary information. RNA-seq data and ChIP-seq data in this paper have been deposited in the NCBI database (BioProject: PRJNA913404).
