# Supplementary figures and images for: Wdr5-mediated H3K4me3 coordinately regulates cell differentiation, proliferation termination, and survival in digestive organogenesis
Source: Cell Death Discov. 2023 Jul 5;9:227. doi: 10.1038/s41420-023-01529-4 (PMC10323123; doi:10.1038/s41420-023-01529-4)

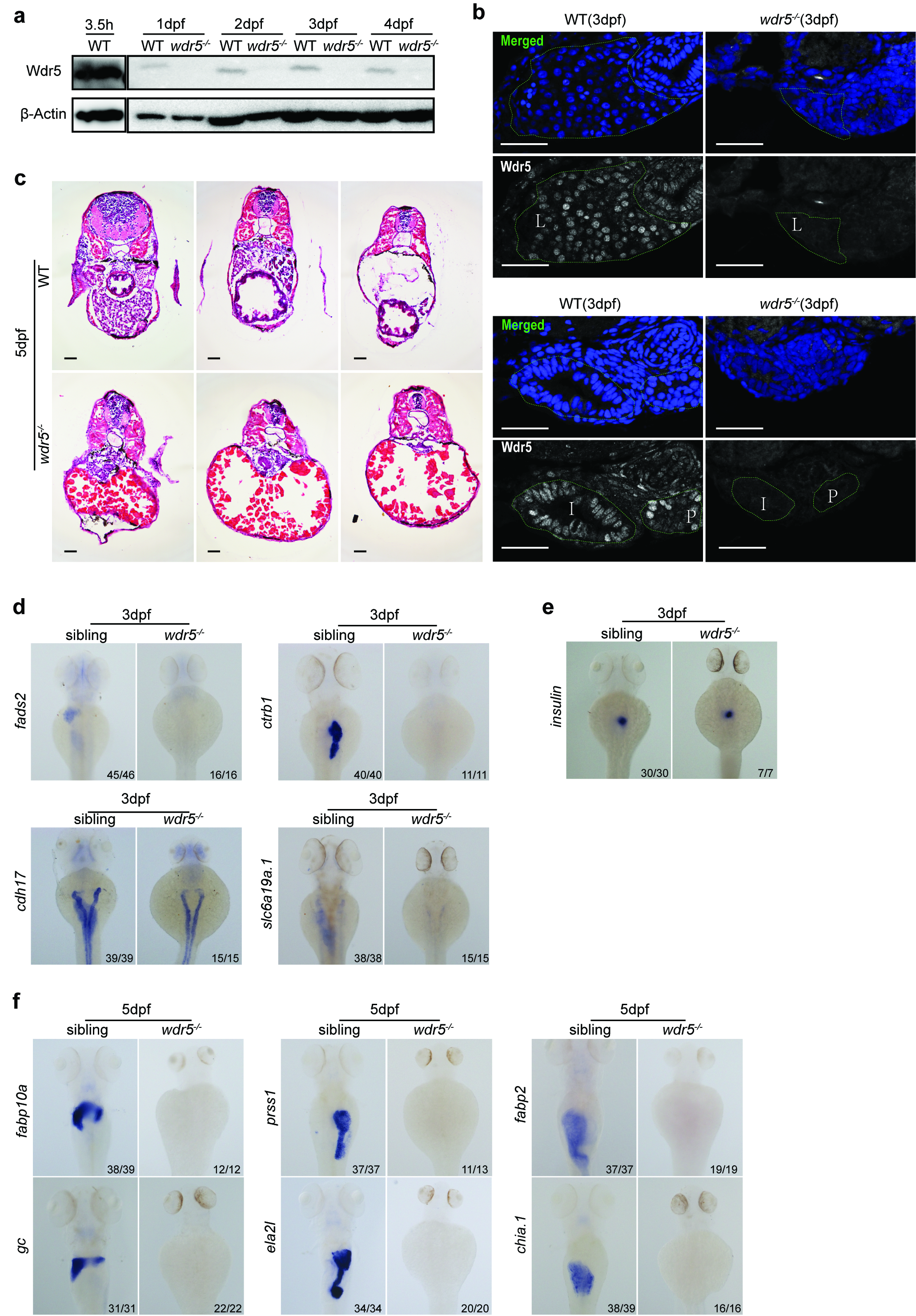

Supplement: Supplementary file 1 — Supplementary Fig 1 [file 41420_2023_1529_MOESM1_ESM.tif]

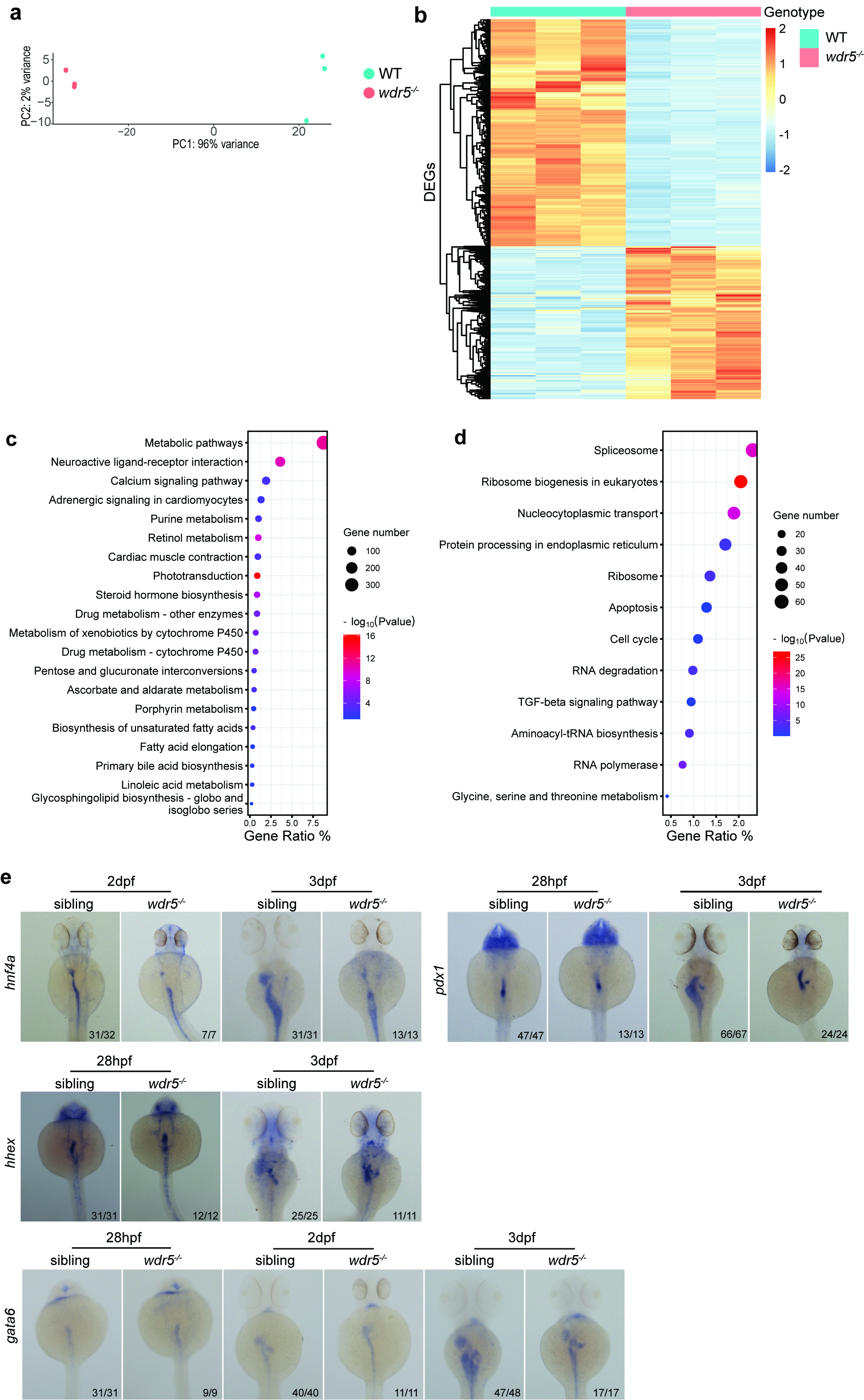

Supplement: Supplementary file 2 — Supplementary Fig 2 [file 41420_2023_1529_MOESM2_ESM.tif]

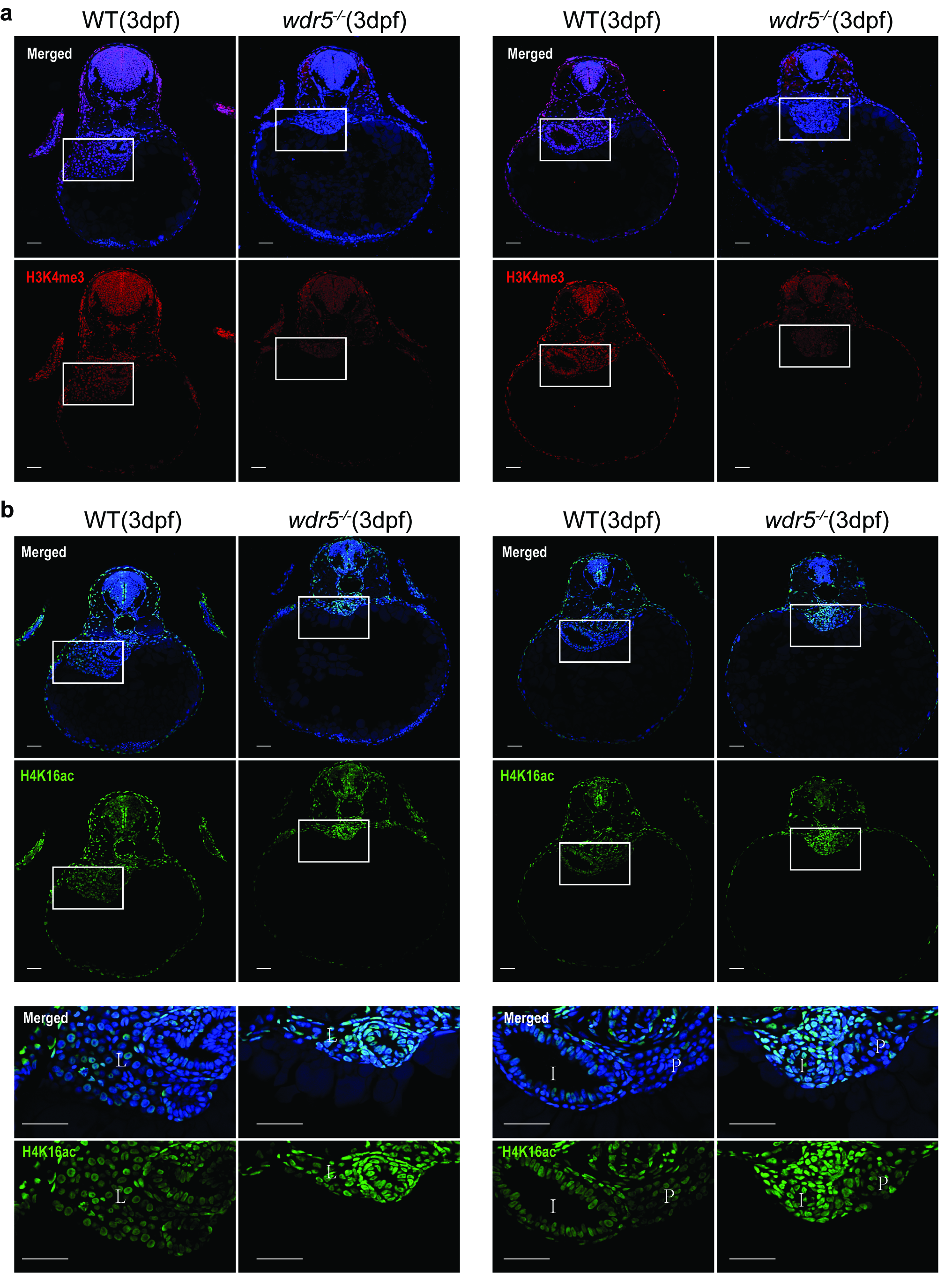

Supplement: Supplementary file 3 — Supplementary Fig 3 [file 41420_2023_1529_MOESM3_ESM.tif]

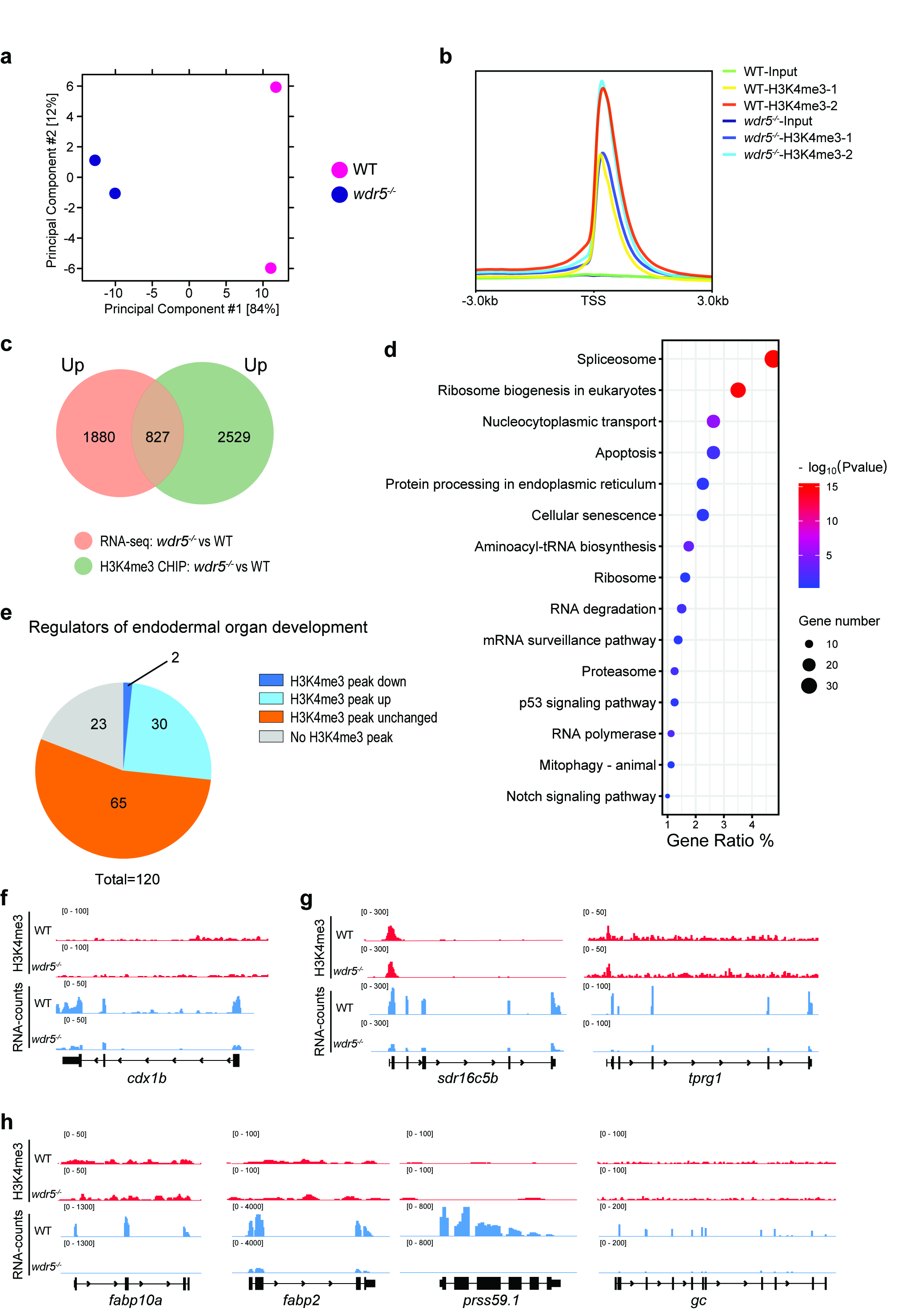

Supplement: Supplementary file 4 — Supplementary Fig 4 [file 41420_2023_1529_MOESM4_ESM.tif]

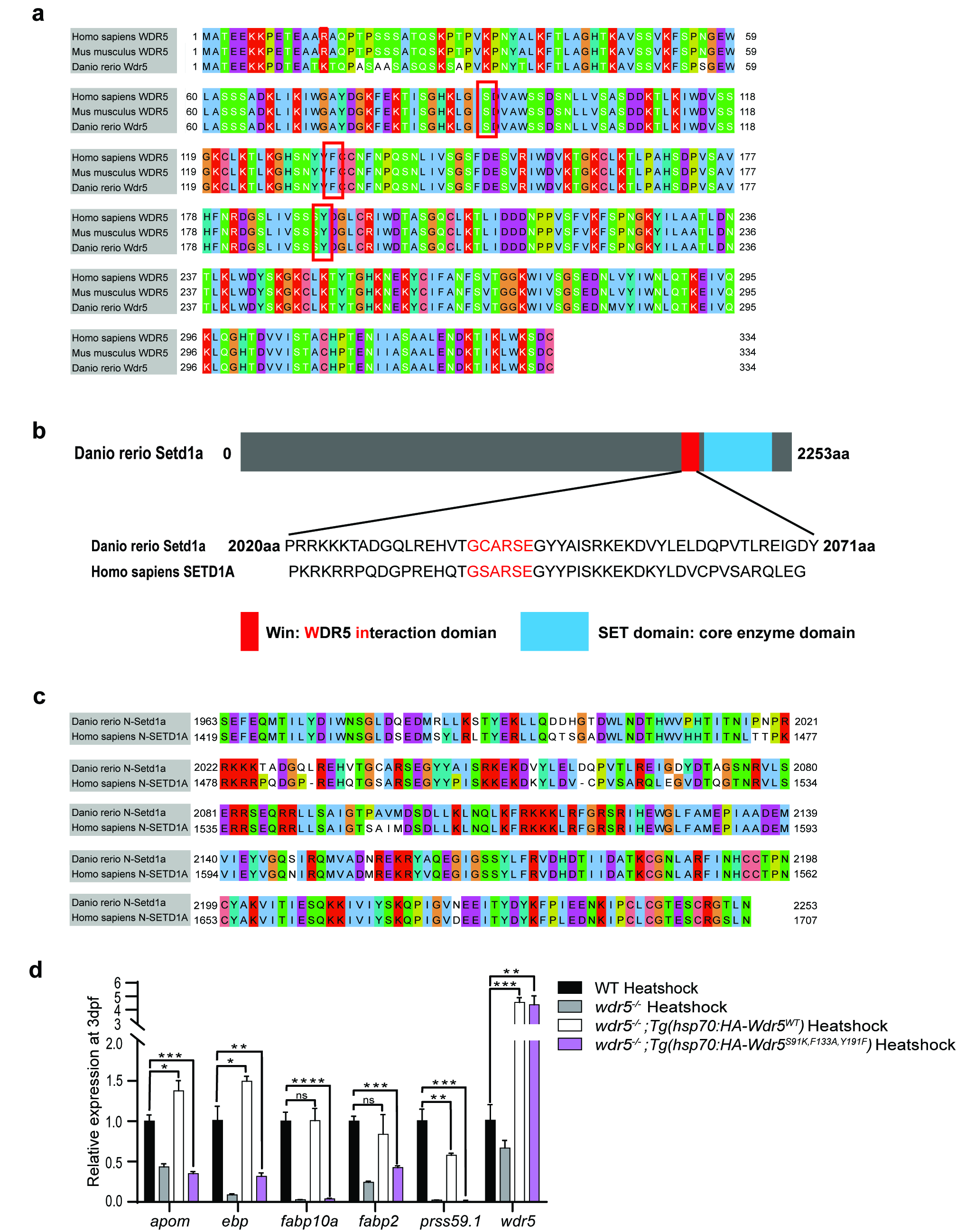

Supplement: Supplementary file 5 — Supplementary Fig 5 [file 41420_2023_1529_MOESM5_ESM.tif]

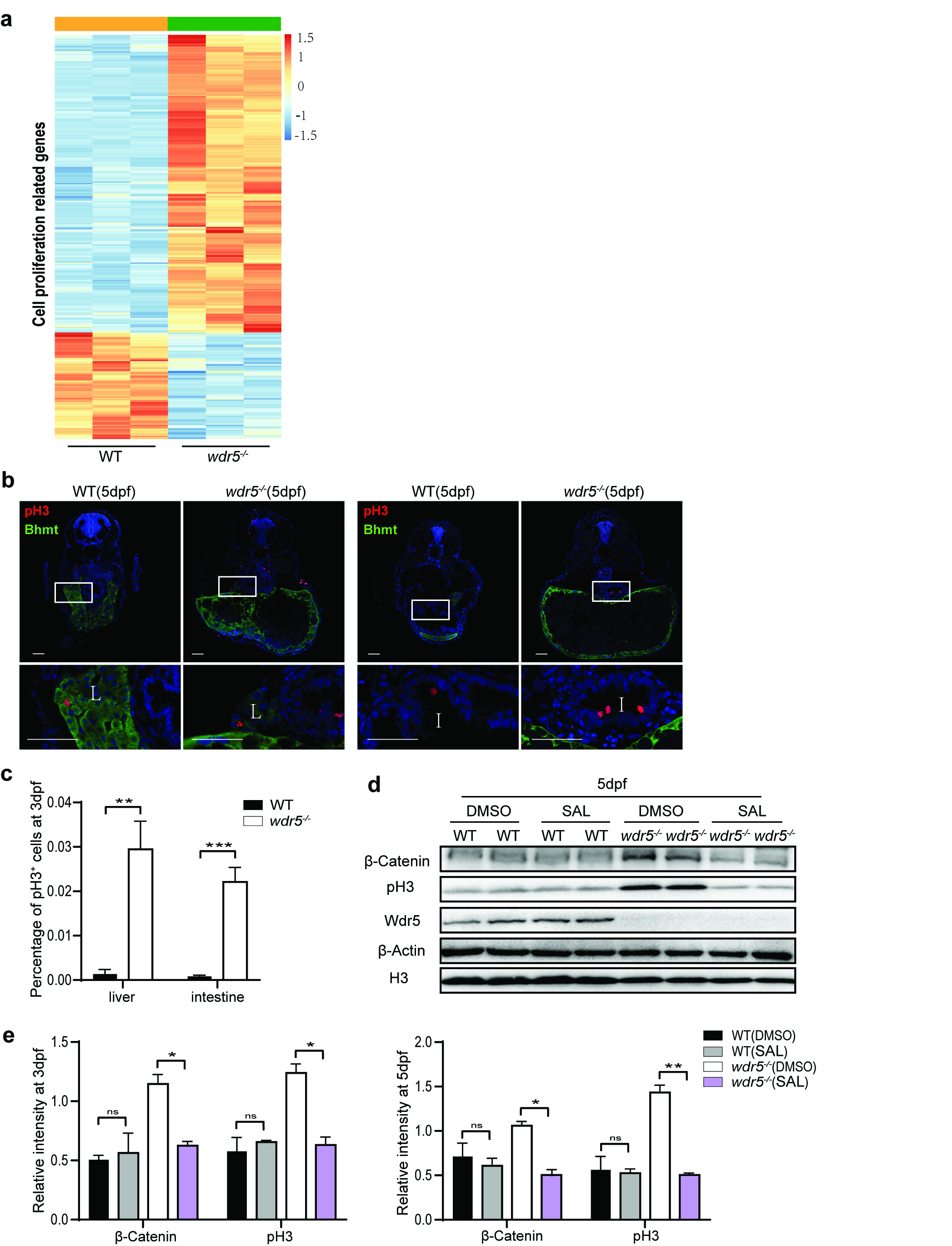

Supplement: Supplementary file 6 — Supplementary Fig 6 [file 41420_2023_1529_MOESM6_ESM.tif]

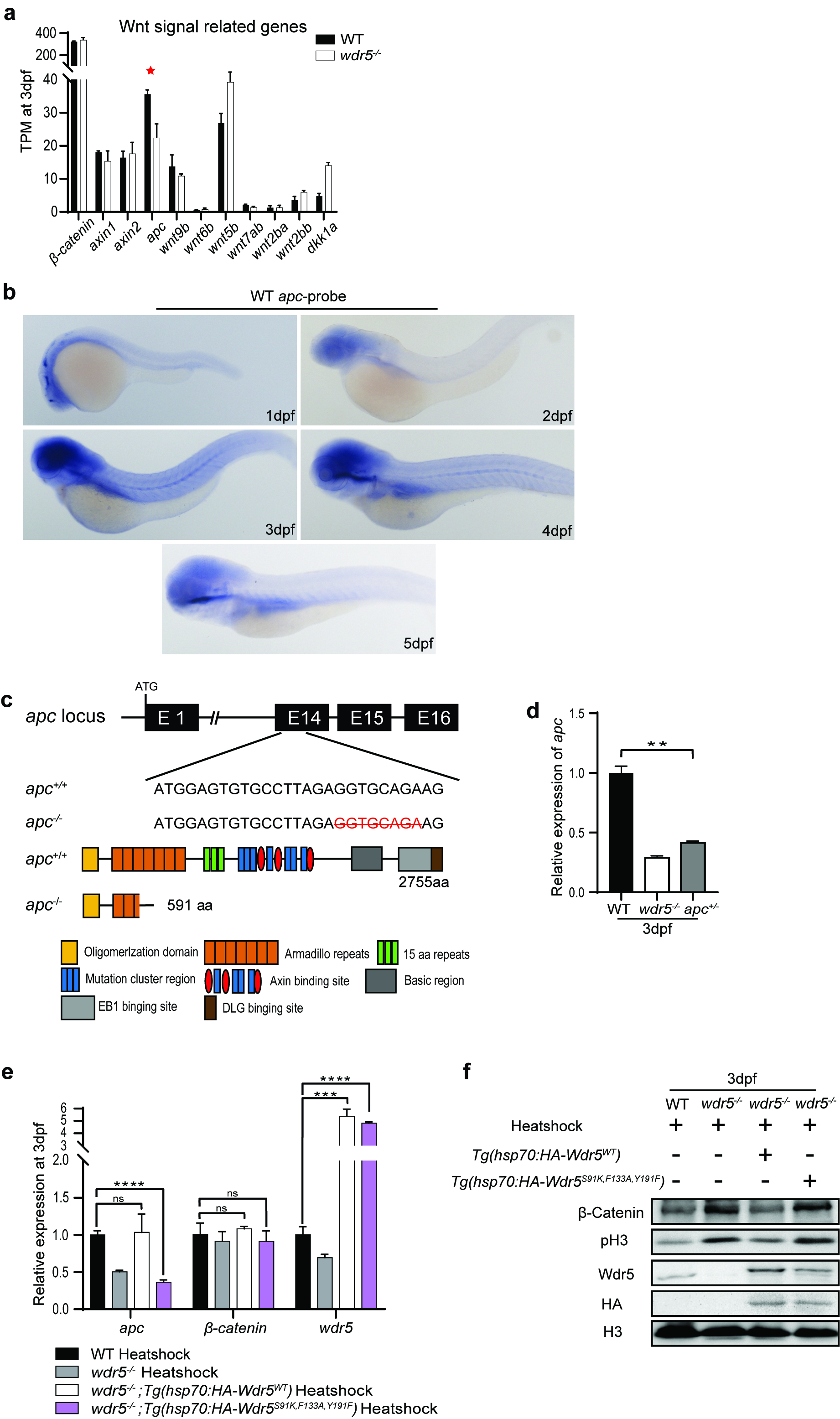

Supplement: Supplementary file 7 — Supplementary Fig 7 [file 41420_2023_1529_MOESM7_ESM.tif]

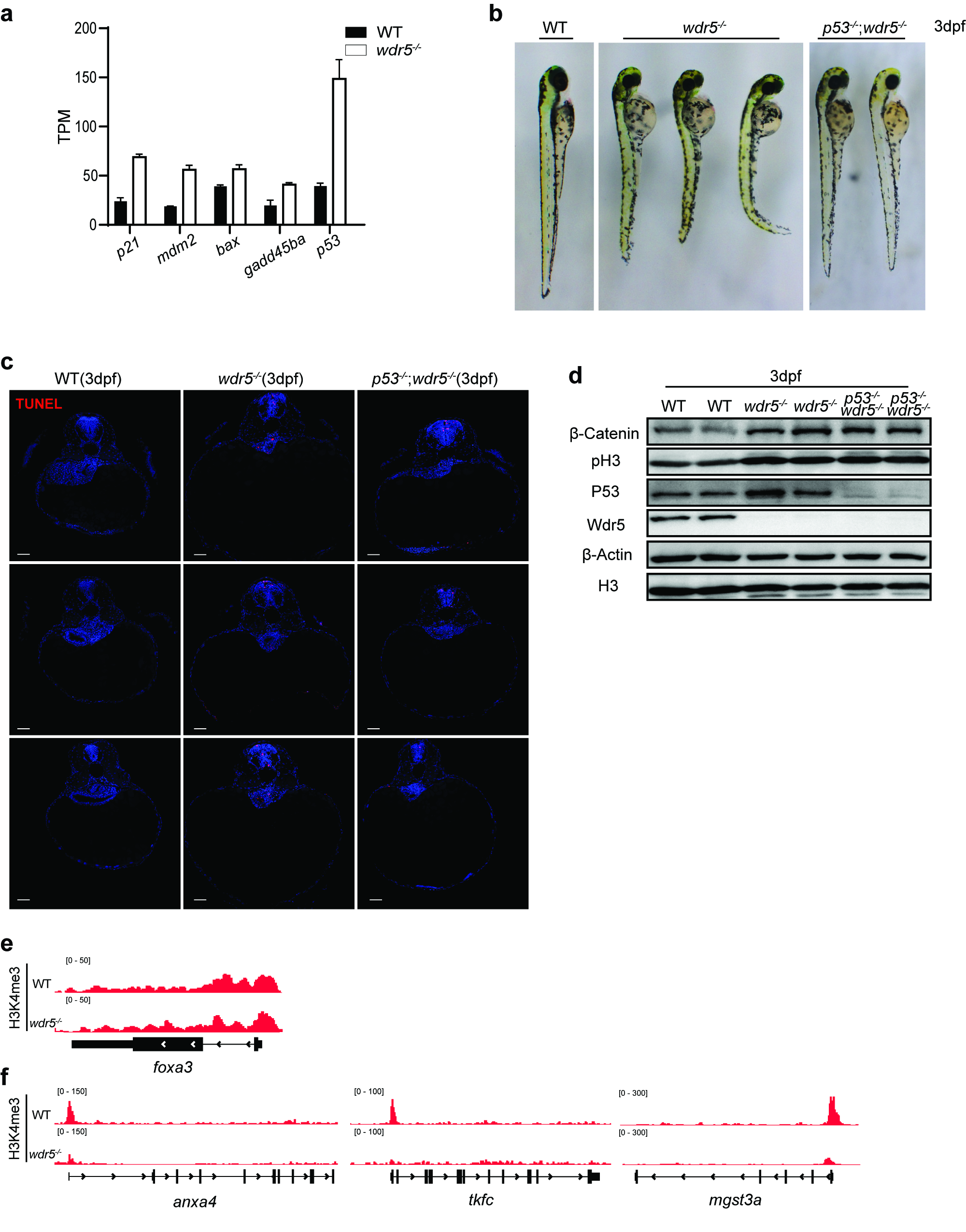

Supplement: Supplementary file 8 — Supplementary Fig 8 [file 41420_2023_1529_MOESM8_ESM.tif]
