# Supplementary material for: Wdr5-mediated H3K4me3 coordinately regulates cell differentiation, proliferation termination, and survival in digestive organogenesis
Source: Cell Death Discov. 2023 Jul 5;9:227. doi: 10.1038/s41420-023-01529-4 (PMC10323123; doi:10.1038/s41420-023-01529-4)
Supplement: Supplementary file 9 — supplementary figure legends [file 41420_2023_1529_MOESM9_ESM.docx]

**Supplementary information**

There are 8 Supplementary Figure Legends and 7 Supplementary Tables in this files

**Supplementary Fig. 1**

**The differentiation of intestine, liver and exocrine-pancreas is impaired in *wdr5^-/-^* mutant embryos.** **a,** Western blots of Wdr5 and β-Actin in WT at different developmental stages. **b,** Cryosections of WT and *wdr5^-/-^*mutant embryos at 3 dpf were immunostained by anti-Wdr5 (in white). The nuclear was stained with DAPI (in blue). L: liver; I: intestine; P: pancreas. Scale bar: 40 μm. **c,** Hematoxylin-Eosin (H&E) staining of cryosections in WT and *wdr5^-/-^* mutant embryos at 5 dpf. Left: mainly for liver; middle and right mainly for pancreas and intestine. Scale bar: 40 μm. **d,** WISH of *fads2, ctrb1, cdh17* and *slc6a19a.1* in WT and *wdr5^-/-^* mutant embryos at 3 dpf. **e,** WISH of *insulin* in WT and *wdr5^-/-^* mutant embryos at 3 dpf. **f,** WISH of *fabp10a, gc, prss1, ela2l, fabp2* and *chia.1* in WT and *wdr5^-/-^* mutant embryos at 5 dpf.

*n* indicates the number of zebrafish embryos in each group.

**Supplementary Fig. 2**

**Transcriptome analysis of WT and *wdr5^-/-^* mutant embryos at 3 dpf.** **a,** Principal components analysis (PCA). Raw expression data from three independent replicates including WT and *wdr5^-/-^* mutant embryos at 3 dpf, were subjected to PCA. **b,** Heatmap of differential expressed genes (DEGs) between WT and *wdr5^-/-^* mutant at 3 dpf . **c,** Significantly enriched KEGG pathways for down-regulated genes (*wdr5^-/-^* vs WT, log_2_FoldChange ≤ -1, *P_adj_* < 0.05) in *wdr5^-/-^* mutant embryos at 3 dpf. **d,** Significantly enriched KEGG pathways for up-regulated genes (*wdr5^-/-^* vs WT, log_2_FoldChange ≥ 1, *P_adj_* < 0.05) in *wdr5^-/-^* mutant embryos at 3 dpf. **e,** WISH of *hnf4a*, *pdx1*, *hhex* and *gata6* in WT and *wdr5^-/-^* mutant embryos at 28 hpf, 2 or 3 dpf.

*n* indicates the number of zebrafish embryos in each group.

**Supplementary Fig. 3**

**H3K4me3, but not H4K16ac is obviously decreased in the digestive organs of *wdr5^-/-^* mutant embryos at 3 dpf. a,** Cryosections of WT and *wdr5^-/-^*mutant embryos at 3 dpf were immunostained by anti-H3K4me3 (in red). The nuclear was stained with DAPI (in blue). Scale bar: 40 μm. Framed area was magnified **in Fig. 2c**. **b,** Cryosections of WT and *wdr5^-/-^*mutant embryos at 3 dpf were immunostained by anti-H4K16ac (in green). The nuclear was stained with DAPI (in blue). L: liver; I: intestine; P: pancreas. Framed area was magnified in bottom panels. Scale bar: 40 μm.

**Supplementary Fig. 4**

**H3K4me3 ChIP-seq analysis of WT and *wdr5^-/-^* mutant embryos at 3 dpf. a,** Raw H3K4me3 ChIP-seq data from two independent replicates including WT and *wdr5^-/-^* mutant embryos at 3 dpf, were subjected to PCA. **b,** Graph showing the normalized reads distribution profiles of the genome-wide H3K4me3 enrichment in WT and *wdr5^-/-^* mutant embryos at 3 dpf. TSS, transcription start site. **c,** Venn diagram showing 827 overlapping upregulated genes between RNA-seq (*wdr5^-/-^* mutant vs WT, log_2_FoldChange ≥ 1, *P_adj_* < 0.05) and H3K4me3 ChIP-seq (*wdr5^-/-^* mutant vs WT, log_2_FoldChange ≥ 0.58, *P_adj_* < 0.05). **d,** KEGG analysis of overlapping upregulated genes between RNA-seq and H3K4me3 ChIP-seq in **Supplementary Fig. 4c**. **e,** Distribution of H3K4me3 peak changes (*wdr5^-/-^* mutant vs WT, |log_2_FoldChange| ≥0.58, *P_adj_* < 0.05) in 120 regulators involved in digestive organogenesis. **f-h,** Graphs showing H3K4me3 and RNA peaks at *cdx1b* (**f**), *sdr16c5b* and *tprg1* (**g**), *fabp10*, *fabp2*, p*rss59.1* and *gc* (**h**) gene loci in WT and *wdr5^-/-^* mutant embryos at 3 dpf.

**Supplementary Fig. 5**

**The expression of digestive organ differentiation genes in *wdr5^-/-^* mutant embryos is restored by *Tg(hsp70:HA-Wdr5^WT^)*, but not *Tg(hsp70:HA-Wdr5^S91K,F133A,Y191F^)*. a,** Amino acid sequence alignment of Wdr5 proteins between zebrafish (Danio rerio NP_998264) and its orthologs in human (Homo sapiens NP_001371338), mouse (Mus musculus NP_543124). The three residues framed by red squares are key residues for the interaction between WDR5 and H3. **b,** Diagram showing the known domains of zebrafish Setd1a protein and the key residues (in red) of Win motifs (for the interaction with Wdr5) in both zebrafish and human SETD1A. **c,** Amino acid sequence alignment of zebrafish N-Setd1a (1963aa-2253aa) proteins (Danio rerio XP_001920852.4) and its orthologs N-SETD1A (1419aa-1707aa) (Homo sapiens NP_055527.1) in human. **d,** qRT-PCR showing expression of *apom, ebp, fabp10a, fabp2, prss59.1* and *wdr5* in different samples at 3 dpf as indicated. The embryos from *Tg(hsp70:HA-Wdr5^WT^)* or *Tg(hsp70:HA-Wdr5^S91K,F133A,Y191F^)* transgenic line in *wdr5^+/-^* background were treated heat shocks as described in **Fig. 3a**. More than 300 embryos were genotyped from each cross after heat shock treatment**.**

Each experiment was repeated for three times with similar results and a representative was showed here. Data are mean±S.D. Two-tailed *t-*test was applied for each individual comparison (*p<0.05; **p<0.01; ***p<0.001; ****p<0.0001; n.s: no significance).

**Supplementary Fig. 6**

**The increase of cell proliferation in the digestive organs of *wdr5^-/-^* mutant embryos is dependent on the activation of Wnt/β-Catenin signal.** **a,** Heatmap of DEGs related to cell proliferation between WT and *wdr5^-/-^* mutant at 3 dpf. **b,** Cryosections of WT and *wdr5^-/-^*mutant embryos at 5 dpf were immunostained by anti-pH3 (in red) and anti-Bhmt (in green). The nuclear was stained with DAPI (in blue). Scale bar: 40 μm. Framed area was magnified in the corresponding bottom panel. L: liver; I: intestine. **c,** Statistical analysis on the percentages of pH3 positive cells in the liver or intestine between WT and *wdr5^-/-^* mutant at 5 dpf was in **Supplementary Fig. 6b**. **d,** Western blots of β-Catenin, pH3, Wdr5, β-Actin and H3 in WT and *wdr5^-/-^*mutant embryos with different treatment as indicated. The *wdr5^-/-^* mutant and WT embryos at 2.3 or 4 dpf were treated with salinomycin sodium salt (SAL) or DMSO. The protein was extracted from treated embryos at 3 and 5 dpf. Also see **Fig. 4e**. **e,** Relative intensities of β-Catenin and pH3 in **Fig. 4e** (left ) and **Supplementary Fig. 6d** (right) were normalized with H3.

Each experiment was repeated for three times with similar results and a representative was showed here. Data are mean±S.D. Two-tailed *t-*test was applied for each individual comparison (*p<0.05; **p<0.01; ***p<0.001; n.s: no significance).

**Supplementary Fig. 7**

**Wdr5-mediated H3K4me3 inhibits Wnt/β-catenin signaling through upregulating *apc* expression. a,** Transcript TPM of regulators of Wnt/β-Catenin signal in RNA-seq from WT and *wdr5^-/-^*mutant embryos at 3 dpf. **b,** WISH of *apc* in WT embryos at 1-5 dpf. **c,** Diagram showing the gRNA targeting site and 8-bp deletion (in red) in the exon 14 of *apc* mutant, which results in a premature stop codon (PTC) at 591 aa. ATG: translation start codon. Different function domains of Apc were labeled with different color boxes. **d,** Relative expression levels of *apc* in WT, *wdr5^-/-^* mutant and *apc^+/-^* mutant were analyzed by qRT-PCR. **e,** qRT-PCR showing expression of *apc, β-catenin* and *wdr5* in different samples at 3 dpf. The embryos from *Tg(hsp70:HA-Wdr5^WT^)* or *Tg(hsp70:HA-Wdr5^S91K,F133A,Y191F^)* transgenic line in *wdr5^+/-^* background were treated heat shocks as described in **Fig. 3a**. More than 300 embryos were genotyped from each cross after heat shock treatment**.** **f,** Western blots of β-Catenin, pH3, Wdr5, HA and H3 in different samples as indicated. The embryos from *Tg(hsp70:HA-Wdr5^WT^)* or *Tg(hsp70:HA-Wdr5^S91K,F133A,Y191F^)* transgenic line in *wdr5^+/-^* background were treated heat shocks as described in **Fig. 3a**. More than 200 embryos were genotyped from each cross after heat shock treatment**.**

Each experiment was repeated for three times with similar results and a representative was showed here. Data are mean±S.D. Two-tailed *t-*test was applied for each individual comparison (**p<0.01; ***p<0.001; ****p<0.0001; n.s: no significance).

**Supplementary Fig. 8**

**The increase of apoptotic activity in *wdr5^-/-^* mutant is largely dependent on the activation of p53. a,** Transcript TPM of p53-target genes in RNA-seq from WT and *wdr5^-/-^*mutant embryos at 3 dpf. **b,** Representative images from WT, *wdr5^-/-^*and *p53^-/-^;wdr5^-/-^* embryos at 3 dpf. **c,** Cryosections of WT, *wdr5^-/-^* and *p53^-/-^;wdr5^-/-^* embryos at 3 dpf were analyzed by TUNEL assay (in red). The nuclear was stained with DAPI (in blue). **d,** Western blots of β-Catenin, pH3, Wdr5, p53, β-Actin and H3 in WT, wdr5^-/-^and *p53^-/-^;wdr5^-/-^* mutant embryos at 3 dpf. **e, f,** Graphs showing H3K4me3 and RNA peaks at *foxa3* (**e**), *anxa4*, *tkfc* and *mgst3a* (**f**) gene loci in WT and *wdr5^-/-^* mutant embryos at 3 dpf.

**Supplementary Table S1. Summary of primer sequences for different experimental designs**

| **Primers for clonning *wdr5* cDNA** | |
| --- | --- |
| wdr5 atg F | atggcaactgaggagaagaa |
| wdr5 tga R | ttagcagtcgcttttccaaa |
| **Primers for WISH probes** | |
| wdr5 antisense probe F | agcacctgtcaagccaaact |
| wdr5 antisense probe R | tggcagccaggatgtacttg |
| apc antisense probe F | agcaatctctgacaaggccc |
| apc antisense probe R | aacaagtccaggcttctccg |
| zgc:171740 antisense probe F | tttgagcgggcagacttgat |
| zgc:171741 antisense probe R | agttcacaaagttgcgtgcc |
| cdh17 antisense probe F | ggctgtacttggagcaacct |
| cdh17 antisense probe R | cccgtgtcgtactgctcaat |
| ela2l antisense probe F | gttctggttgtcggagcgta |
| ela2l antisense probe R | tatcggccaggggtccatta |
| apom antisense probe F | ataccggcacttggtatt |
| apom antisense probe R | agcaaacacag |
| ebp antisense probe F | ccaatggaggcagaagctgt |
| ebp antisense probe R | accgtacagctgacccaatg |
| fads2 antisense probe F | gacagaccgaatcaccgaca |
| fads2 antisense probe R | gcaaccatccagcttgtga |
| ctrb1 antisense probe F | gtggatcctctcctgcctcg |
| ctrb1 antisense probe R | ggttccccagtaacgcttg |
| anxa4 antisense probe F | acatgagctgagaaacgcca |
| anxa4 antisense probe R | ctatctctgcacgagccacc |
| mgst3a antisense probe F | cggttgctagcttagaggtc |
| mgst3a antisense probe R | ccatccgagcatcactctcc |
| fads2 antisense probe F | gacagaccgaatcaccgaca |
| fads2 antisense probe R | gcaaccatccagcttgtga |
| chia.1 antisense probe F | gcaattgaacaagaggccatcg |
| chia.1 antisense probe R | aacagaagcggccagc |
| tkfc antisense probe F | caggagtggctccagacaag |
| tkfc antisense probe R | taaagcatcgagtacggccc |
| **Primers for real time quantitative PCR (qPCR)** | |
| apc F | gctctgataaagtgtgcctt |
| apc R | ctgtgctcttcgtttgtggc |
| myca F | gcaaggttgcatcaccacag |
| myca R | gactggggtacctcgactct |
| ccnd1 F | gaagtgggatctggcctcag |
| ccnd1 R | atgagaggcaactgtcggtg |
| p53 e1-4 F | cgcggatttgctttgtggat |
| p53 e1-4 R | gggatagtcgcttgtctccg |
| p53 e5-12 F | aagagtggagggcaatcagc |
| p53 e5-12 R | tacgtttggtcccagtggtg |
| wdr5 F | aagacgctgaagggacacag |
| wdr5 R | ctggtgggttgtcgtcatca |
| ctnnd1 F | agtcgagtgctcaaggtgtt |
| ctnnd1 R | ccctccatgccctcctgttt |
| actin F | gatgcccctcgtgctgttttc |
| actin R | tctctgttggctttgggattca |
| **Primers for plasmid construction** | |
| BamhⅠ-kozak-HA-wdr5 F | tacttgttctttttgcaggccaccatgtacccatacgatgttcc |
| BamhⅠ-wdr5 R | ttgaattcgaatcgatggttagcagtcgcttttccaaa |
| NotⅠ-kozak-HA-wdr5 F | ttgaaatcaatctctagagcgccaccatgtacccatacgatgttcc |
| wdr5 fragment1(S91K) R | gctacatctttgatgccaag |
| wdr5 fragment2(S91K) F | cttggcatcaaagatgtagc |
| wdr5 fragment2(F133A) R | tacagcaggccacataattg |
| wdr5 fragment3(F133A) F | caattatgtggcctgctgta |
| wdr5 fragment3(Y191F) R | gtccatcaaaactgctggac |
| wdr5 fragment4(Y191F) F | gtccagcagttttgatggac |
| NotⅠ-polyA R | cccaatgcattggcgccgcgaattaaaaaacctcccaca |
| BamhⅠ-kozak-Flag-N-setd1a F | tacttgttctttttgcaggccaccatggactacaaggacgacgatgacaaaagcgagtttgagcagat |
| BamhⅠ- setd1a R | ttgaattcgaatcgatggttagttgagggttcctctg |
| **Primers for genotyping mutant and transgenetic zebrafish** | |
| wdr5 mutant ID F | ttctctccaacttggtcgcc |
| wdr5 mutant ID R | ttaccttactctggctggca |
| hsp70-wdr5 transgenic ID F | tagagcgccaccatgtaccc |
| hsp70-wdr5 transgenic ID R | actccccacttggactgaattt |
| p53 mutant ID F | ctgtcagctggcaaaaacttg |
| p53 mutant ID R | cacatgctcggacttcttatag |
| apc mutant ID F | ctctcatggcgagctgatgt |
| apc mutant ID R | caaagggcactcaggacact |
| zgc:171740 mutant ID F | tgttctcacagatgtgtccatt |
| zgc:171740 mutant ID R | tgagggcttcagattcagcta |
| **Primers for generating mutant** | |
| zgc:171740 guide-RNA target sequence | ggctgaaggtgaaggacctgcgg |
| zgc:171740 guide-RNA PCR F | ataatacgactcactataggctgaaggtgaaggacctggttttagagctagaaatagc |
| p53 guide-RNA target sequence | gtggacgttgcccctccacagg |
| p53 guide-RNA PCR F | ataatacgactcactatagtggacgttgcccctccacgttttagagctagaaatagc |
| apc guide-RNA target sequence | gtgtgccttagaggtgcagaagg |
| apc guide-RNA PCR F | ataatacgactcactatagtgtgccttagaggtgcagagttttagagctagaaatagc |
| guide-RNA PCR R | agcaccgactcggtgccact |
| zgc:171740 F0 genotyping F | tgttctcacagatgtgtccatt |
| zgc:171740 F0 genotyping R | tgagggcttcagattcagcta |
| p53 F0 genotyping F | ctgtcagctggcaaaaacttg |
| p53 F0 genotyping R | cacatgctcggacttcttatag |
| apc F0 genotyping F | gttcaatacttatcttccctgctg |
| apc F0 genotyping R | tgttggtttggcttcggt |

**Supplementary Table S2. Statistical anlysis of pH3 postive cells in the endodermal organs**

| **Figure. 4b** | | | | | | |
| --- | --- | --- | --- | --- | --- | --- |
| liver | positive cells | total cells | percentage | average | Stdev | T-test |
| wt1 | 26 | 1006 | 0.02584 | 0.0352 | 0.00928 | 0.03963 |
| wt2 | 34 | 972 | 0.03498 |  |  |  |
| wt3 | 27 | 913 | 0.02957 |  |  |  |
| wt4 | 32 | 904 | 0.0354 |  |  |  |
| wt5 | 24 | 478 | 0.05021 |  |  |  |
| mu1 | 5 | 121 | 0.04132 | 0.05271 | 0.01162 |  |
| mu2 | 1 | 17 | 0.05882 |  |  |  |
| mu3 | 3 | 67 | 0.04478 |  |  |  |
| mu4 | 6 | 91 | 0.06593 |  |  |  |
| intestine | positive cells | total cells | percentage | average | Stdev | T-test |
| wt1 | 39 | 706 | 0.05524 | 0.05044 | 0.0068 | 0.041 |
| wt2 | 17 | 327 | 0.05199 |  |  |  |
| wt3 | 31 | 581 | 0.05336 |  |  |  |
| wt4 | 6 | 156 | 0.03846 |  |  |  |
| wt5 | 26 | 489 | 0.05317 |  |  |  |
| mu1 | 13 | 146 | 0.08904 | 0.08084 | 0.0276 |  |
| mu2 | 8 | 107 | 0.07477 |  |  |  |
| mu3 | 19 | 155 | 0.12258 |  |  |  |
| mu4 | 5 | 108 | 0.0463 |  |  |  |
| mu5 | 8 | 84 | 0.09524 |  |  |  |
| mu6 | 6 | 105 | 0.05714 |  |  |  |
| **Figure. 5d** | | | | | | |
| liver | positive cells | total cells | percentage | average | Stdev | T-test |
| wt1 | 16 | 406 | 0.03941 | 0.03584 | 0.00668 | 0.02288 |
| wt2 | 15 | 506 | 0.02964 |  |  |  |
| wt3 | 34 | 788 | 0.04315 |  |  |  |
| wt4 | 18 | 747 | 0.0241 |  |  |  |
| wt5 | 20 | 500 | 0.04 |  |  |  |
| wt6 | 13 | 336 | 0.03869 |  |  |  |
| wt7 | 28 | 780 | 0.0359 |  |  |  |
| hetro1 | 31 | 509 | 0.0609 | 0.04866 | 0.01116 |  |
| hetro2 | 29 | 444 | 0.06532 |  |  |  |
| hetro3 | 33 | 744 | 0.04435 |  |  |  |
| hetro4 | 36 | 830 | 0.04337 |  |  |  |
| hetro5 | 28 | 852 | 0.03286 |  |  |  |
| hetro6 | 29 | 582 | 0.04983 |  |  |  |
| hetro7 | 31 | 705 | 0.04397 |  |  |  |
| intestine | positive cells | total cells | percentage | average | Stdev | T-test |
| wt1 | 24 | 501 | 0.0479 | 0.04807 | 0.00519 | 0.00011 |
| wt2 | 21 | 456 | 0.04605 |  |  |  |
| wt3 | 22 | 511 | 0.04305 |  |  |  |
| wt4 | 31 | 561 | 0.05526 |  |  |  |
| hetro1 | 41 | 505 | 0.08119 | 0.08071 | 0.00695 |  |
| hetro2 | 36 | 440 | 0.08182 |  |  |  |
| hetro3 | 66 | 727 | 0.09078 |  |  |  |
| hetro4 | 38 | 531 | 0.07156 |  |  |  |
| hetro5 | 38 | 486 | 0.07819 |  |  |  |
| **Supplementary figure. 6b, c** | | | | | | |
| liver | positive cells | total cells | percentage | average | Stdev | T-test |
| wt1 | 1 | 1950 | 0.00615 | 0.00331 | 0.00262 | 0.00272 |
| wt2 | 2 | 2001 | 0.001 |  |  |  |
| wt3 | 5 | 1801 | 0.00278 |  |  |  |
| mu1 | 3 | 99 | 0.0303 | 0.02944 | 0.00633 |  |
| mu2 | 3 | 85 | 0.03529 |  |  |  |
| mu3 | 2 | 88 | 0.02273 |  |  |  |
| intestine | positive cells | total cells | percentage | average | Stdev | T-test |
| wt1 | 2 | 1870 | 0.00107 | 0.00088 | 0.00032 | 0.00036 |
| wt2 | 1 | 1950 | 0.00051 |  |  |  |
| wt3 | 2 | 1881 | 0.00106 |  |  |  |
| mu1 | 6 | 290 | 0.02069 | 0.02212 | 0.00327 |  |
| mu2 | 6 | 303 | 0.0198 |  |  |  |
| mu3 | 9 | 348 | 0.02586 |  |  |  |
| **Figure. 4g** | | | | | | |
| liver | positive cells | total cells | percentage | average | Stdev | T-test |
| mu DMSO1 | 6 | 104 | 0.05769 | 0.06022 | 0.00632 | 0.01943 |
| mu DMSO2 | 6 | 108 | 0.05556 |  |  |  |
| mu DMSO3 | 6 | 89 | 0.06742 |  |  |  |
| mu SAL1 | 1 | 84 | 0.0119 | 0.02706 | 0.01382 |  |
| mu SAL2 | 3 | 77 | 0.03896 |  |  |  |
| mu SAL3 | 3 | 99 | 0.0303 |  |  |  |
| intestine | positive cells | total cells | percentage | average | Stdev | T-test |
| mu DMSO1 | 11 | 218 | 0.05046 | 0.07181 | 0.01863 | 0.01484 |
| mu DMSO2 | 15 | 177 | 0.08475 |  |  |  |
| mu DMSO3 | 15 | 187 | 0.08021 |  |  |  |
| mu SAL1 | 6 | 190 | 0.03158 | 0.02623 | 0.00486 |  |
| mu SAL2 | 4 | 181 | 0.0221 |  |  |  |
| mu SAL3 | 4 | 160 | 0.025 |  |  |  |
| **Figure. 5g** | | | | | | |
| liver | positive cells | total cells | percentage | average | Stdev | T-test |
| wt 3d1 | 26 | 1006 | 0.02584 | 0.0352 | 0.00928 | 0.0009 |
| wt 3d2 | 34 | 972 | 0.03498 |  |  |  |
| wt 3d3 | 27 | 913 | 0.02957 |  |  |  |
| wt 3d4 | 32 | 904 | 0.0354 |  |  |  |
| wt 3d5 | 24 | 478 | 0.05021 |  |  |  |
| wt 5d1 | 1 | 1950 | 0.00051 | 0.00143 | 0.00119 |  |
| wt 5d2 | 2 | 2001 | 0.001 |  |  |  |
| wt 5d3 | 5 | 1801 | 0.00278 |  |  |  |
| intestine | positive cells | total cells | percentage | average | Stdev | T-test |
| wt 3d1 | 39 | 706 | 0.05524 | 0.05044 | 0.0068 | 1.8E-05 |
| wt 3d2 | 17 | 327 | 0.05199 |  |  |  |
| wt 3d3 | 31 | 581 | 0.05336 |  |  |  |
| wt 3d4 | 6 | 156 | 0.03846 |  |  |  |
| wt 3d5 | 26 | 489 | 0.05317 |  |  |  |
| wt 5d1 | 2 | 1870 | 0.00107 | 0.00088 | 0.00032 |  |
| wt 5d2 | 1 | 1950 | 0.00051 |  |  |  |
| wt 5d3 | 2 | 1881 | 0.00106 |  |  |  |
| Note: wt: wild type; mu: *wdr5^-/-^*; hetro: *apc^+/-^* | | | | | | |

**Supplementary Table S3. Statistical anlysis of cell number in liver**

| **Figure. 6e** | | | | |
| --- | --- | --- | --- | --- |
| genotype | liver cell number | average | Stdev | T-test |
| wt1 | 1006 | 963.667 | 47.0567 | 7.9E-06 |
| wt2 | 972 |  |  |  |
| wt3 | 913 |  |  |  |
| mu1 | 138 | 122 | 15.5242 |  |
| mu2 | 107 |  |  |  |
| mu3 | 121 |  |  |  |
| genotype | liver cell number | average | Stdev | T-test |
| mu1 | 138 | 122 | 15.5242 | 0.00247 |
| mu2 | 107 |  |  |  |
| mu3 | 121 |  |  |  |
| dm1 | 350 | 322.333 | 48.7887 |  |
| dm2 | 266 |  |  |  |
| dm3 | 351 |  |  |  |
| Note: wt: wild type; mu: *wdr5^-/-^*; dm: *p53^-/-^;wdr5^-/-^* | | | | |

**Supplementary Table S4. Statistical anlysis of apoptoic cell ratio in wild type and mutant**

| **Figure. 6d** | | | | | | |
| --- | --- | --- | --- | --- | --- | --- |
| liver | apoptoic cells | total cells | percentage | average | Stdev | T-test |
| wt1 | 0 | 135 | 0 | 0.00226 | 0.00311 | 2.7E-09 |
| wt2 | 3 | 393 | 0.00763 |  |  |  |
| wt3 | 0 | 314 | 0 |  |  |  |
| wt4 | 0 | 482 | 0 |  |  |  |
| wt5 | 0 | 651 | 0 |  |  |  |
| wt6 | 2 | 390 | 0.00513 |  |  |  |
| wt7 | 2 | 656 | 0.00305 |  |  |  |
| mu1 | 17 | 196 | 0.08673 | 0.07669 | 0.0135 |  |
| mu2 | 6 | 62 | 0.09677 |  |  |  |
| mu3 | 6 | 85 | 0.07059 |  |  |  |
| mu4 | 4 | 75 | 0.05333 |  |  |  |
| mu5 | 7 | 99 | 0.07071 |  |  |  |
| mu6 | 8 | 97 | 0.08247 |  |  |  |
| mu7 | 8 | 95 | 0.08421 |  |  |  |
| mu8 | 9 | 131 | 0.0687 |  |  |  |
| pancreas | apoptoic cells | total pixels | percentage | average | Stdev | T-test |
| wt1 | 1 | 121 | 0.00826 | 0.00432 | 0.00474 | 7.5E-07 |
| wt2 | 3 | 246 | 0.0122 |  |  |  |
| wt3 | 0 | 224 | 0 |  |  |  |
| wt4 | 2 | 363 | 0.00551 |  |  |  |
| wt5 | 0 | 380 | 0 |  |  |  |
| wt6 | 0 | 339 | 0 |  |  |  |
| wt7 | 2 | 465 | 0.0043 |  |  |  |
| mu1 | 11 | 114 | 0.09649 | 0.07559 | 0.02081 |  |
| mu2 | 15 | 161 | 0.09317 |  |  |  |
| mu3 | 13 | 155 | 0.08387 |  |  |  |
| mu4 | 7 | 129 | 0.05426 |  |  |  |
| mu5 | 7 | 178 | 0.03933 |  |  |  |
| mu6 | 12 | 127 | 0.09449 |  |  |  |
| mu7 | 10 | 151 | 0.06623 |  |  |  |
| mu8 | 11 | 143 | 0.07692 |  |  |  |
| intestine | apoptoic cells | total cells | percentage | average | Stdev | T-test |
| wt1 | 1 | 264 | 0.00379 | 0.0009 | 0.00158 | 1E-09 |
| wt2 | 0 | 340 | 0 |  |  |  |
| wt3 | 0 | 276 | 0 |  |  |  |
| wt4 | 0 | 476 | 0 |  |  |  |
| wt5 | 1 | 398 | 0.00251 |  |  |  |
| wt6 | 0 | 672 | 0 |  |  |  |
| wt7 | 0 | 821 | 0 |  |  |  |
| mu1 | 6 | 118 | 0.05085 | 0.0654 | 0.01094 |  |
| mu2 | 11 | 176 | 0.0625 |  |  |  |
| mu3 | 5 | 87 | 0.05747 |  |  |  |
| mu4 | 12 | 190 | 0.06316 |  |  |  |
| mu5 | 12 | 159 | 0.07547 |  |  |  |
| mu6 | 13 | 227 | 0.05727 |  |  |  |
| mu7 | 10 | 120 | 0.08333 |  |  |  |
| mu8 | 9 | 123 | 0.07317 |  |  |  |
| liver | apoptoic cells | total cells | percentage | average | Stdev | T-test |
| dm1 | 4 | 162 | 0.02469 | 0.0162 | 0.00772 | 4.6E-07 |
| dm2 | 2 | 278 | 0.00719 |  |  |  |
| dm3 | 2 | 222 | 0.00901 |  |  |  |
| dm4 | 7 | 280 | 0.025 |  |  |  |
| dm5 | 2 | 155 | 0.0129 |  |  |  |
| dm6 | 5 | 272 | 0.01838 |  |  |  |
| mu1 | 17 | 196 | 0.08673 | 0.07669 | 0.0135 |  |
| mu2 | 6 | 62 | 0.09677 |  |  |  |
| mu3 | 6 | 85 | 0.07059 |  |  |  |
| mu4 | 4 | 75 | 0.05333 |  |  |  |
| mu5 | 7 | 99 | 0.07071 |  |  |  |
| mu6 | 8 | 97 | 0.08247 |  |  |  |
| mu7 | 8 | 95 | 0.08421 |  |  |  |
| mu8 | 9 | 131 | 0.0687 |  |  |  |
| pancreas | apoptoic cells | total pixels | percentage | average | Stdev | T-test |
| dm1 | 4 | 119 | 0.03361 | 0.0248 | 0.0055 | 8.8E-05 |
| dm2 | 5 | 204 | 0.02451 |  |  |  |
| dm3 | 4 | 215 | 0.0186 |  |  |  |
| dm4 | 5 | 209 | 0.02392 |  |  |  |
| dm5 | 3 | 150 | 0.02 |  |  |  |
| dm6 | 6 | 213 | 0.02817 |  |  |  |
| mu1 | 11 | 114 | 0.09649 | 0.07559 | 0.02081 |  |
| mu2 | 15 | 161 | 0.09317 |  |  |  |
| mu3 | 13 | 155 | 0.08387 |  |  |  |
| mu4 | 7 | 129 | 0.05426 |  |  |  |
| mu5 | 7 | 178 | 0.03933 |  |  |  |
| mu6 | 12 | 127 | 0.09449 |  |  |  |
| mu7 | 10 | 151 | 0.06623 |  |  |  |
| mu8 | 11 | 143 | 0.07692 |  |  |  |
| intestine | apoptoic cells | total cells | percentage | average | Stdev | T-test |
| dm1 | 6 | 294 | 0.02041 | 0.01742 | 0.00654 | 6.3E-07 |
| dm2 | 2 | 105 | 0.01905 |  |  |  |
| dm3 | 1 | 97 | 0.01031 |  |  |  |
| dm4 | 2 | 156 | 0.01282 |  |  |  |
| dm5 | 2 | 146 | 0.0137 |  |  |  |
| dm6 | 9 | 319 | 0.02821 |  |  |  |
| mu1 | 6 | 118 | 0.05085 | 0.0654 | 0.01094 |  |
| mu2 | 11 | 176 | 0.0625 |  |  |  |
| mu3 | 5 | 87 | 0.05747 |  |  |  |
| mu4 | 12 | 190 | 0.06316 |  |  |  |
| mu5 | 12 | 159 | 0.07547 |  |  |  |
| mu6 | 13 | 227 | 0.05727 |  |  |  |
| mu7 | 10 | 120 | 0.08333 |  |  |  |
| mu8 | 9 | 123 | 0.07317 |  |  |  |
| Note: wt: wild type; mu: *wdr5^-/-^*; dm: *p53^-/-^;wdr5^-/-^* | | | | | | |
| **Figure. 7f, g** | | | | | | |
| intestine | apoptoic cells | total cells | percentage | average | Stdev | T-test |
| wt1 | 5 | 1508 | 0.00332 | 0.00693 | 0.00293 | 0.00078 |
| wt2 | 9 | 1760 | 0.00511 |  |  |  |
| wt3 | 12 | 1419 | 0.00846 |  |  |  |
| wt4 | 14 | 1287 | 0.01088 |  |  |  |
| wt5 | 12 | 1746 | 0.00687 |  |  |  |
| mu1 | 33 | 1622 | 0.02035 | 0.03401 | 0.01117 |  |
| mu2 | 76 | 1488 | 0.05108 |  |  |  |
| mu3 | 24 | 777 | 0.03089 |  |  |  |
| mu4 | 35 | 964 | 0.03631 |  |  |  |
| mu5 | 49 | 1558 | 0.03145 |  |  |  |
| Note: wt: wild type; mu: *zgc:171740^-/-^* | | | | | | |

**Supplementary Table S5. Genes related to endodermal organ differentiation**

| **Gene** | **GeneName** | **Gene** | **GeneName** |
| --- | --- | --- | --- |
| ENSDARG00000076448 | serpinf2a | ENSDARG00000038618 | cpt2 |
| ENSDARG00000092044 | si:dkey-22f5.9 | ENSDARG00000037012 | slc3a2b |
| ENSDARG00000046142 | si:ch211-170d8.5 | ENSDARG00000077988 | abcc10 |
| ENSDARG00000007024 | uox | ENSDARG00000088357 | acadl |
| ENSDARG00000038439 | fabp10a | ENSDARG00000027183 | namptb |
| ENSDARG00000095512 | rca2.2 | ENSDARG00000003084 | spire2 |
| ENSDARG00000053480 | aqp9b | ENSDARG00000006760 | slc24a3 |
| ENSDARG00000019492 | shbg | ENSDARG00000005891 | cyb5r3 |
| ENSDARG00000091800 | zgc:174260 | ENSDARG00000009901 | slc38a5a |
| ENSDARG00000070960 | si:ch211-288g17.4 | ENSDARG00000038901 | tmem256 |
| ENSDARG00000001767 | slc29a2 | ENSDARG00000070874 | asmt2 |
| ENSDARG00000056498 | crp2 | ENSDARG00000058005 | hgd |
| ENSDARG00000037191 | ttr | ENSDARG00000101379 | mfsd1 |
| ENSDARG00000053476 | lipca | ENSDARG00000010415 | sirt4 |
| ENSDARG00000045306 | slc51a | ENSDARG00000031814 | dhrs13b |
| ENSDARG00000042613 | crp3 | ENSDARG00000094557 | nupr1b |
| ENSDARG00000003523 | itln3 | ENSDARG00000027750 | dpp7 |
| ENSDARG00000099633 | sec14l7 | ENSDARG00000077872 | CR626907.1 |
| ENSDARG00000105151 | cyp2k21 | ENSDARG00000090722 | leg1.1 |
| ENSDARG00000038666 | igfbp1b | ENSDARG00000102808 | calr3b |
| ENSDARG00000056744 | ela2 | ENSDARG00000098024 | si:dkey-262k9.2 |
| ENSDARG00000021172 | cyp2ad2 | ENSDARG00000014053 | olfm1b |
| ENSDARG00000044387 | cldn2 | ENSDARG00000063101 | man2a2 |
| ENSDARG00000086523 | ch25hl3 | ENSDARG00000035652 | sat1a.1 |
| ENSDARG00000071076 | ldhbb | ENSDARG00000052942 | aspg |
| ENSDARG00000079307 | si:dkey-205h13.1 | ENSDARG00000104108 | slco1d1 |
| ENSDARG00000089310 | gc | ENSDARG00000088140 | hsd17b7 |
| ENSDARG00000101861 | cyp2k19 | ENSDARG00000103025 | hmgcs1 |
| ENSDARG00000079805 | tagln3a | ENSDARG00000011170 | ndrg2 |
| ENSDARG00000077960 | si:ch211-186e20.7 | ENSDARG00000093774 | rbp2b |
| ENSDARG00000005616 | bfb | ENSDARG00000068006 | gck |
| ENSDARG00000018478 | agxtb | ENSDARG00000100709 | agxt2 |
| ENSDARG00000097725 | si:dkey-96g2.1 | ENSDARG00000018351 | hpda |
| ENSDARG00000070078 | abcb11b | ENSDARG00000095751 | leg1.2 |
| ENSDARG00000076773 | lrrc58a | ENSDARG00000079544 | si:ch1073-464p5.5 |
| ENSDARG00000075015 | soul5 | ENSDARG00000029497 | tfcp2l1 |
| ENSDARG00000057741 | nr1h4 | ENSDARG00000016771 | tfa |
| ENSDARG00000069095 | gls2a | ENSDARG00000045888 | acat1 |
| ENSDARG00000008457 | faah2a | ENSDARG00000021869 | rcan2 |
| ENSDARG00000001898 | manea | ENSDARG00000071579 | urad |
| ENSDARG00000041595 | ces3 | ENSDARG00000035569 | cyp1d1 |
| ENSDARG00000068208 | def8 | ENSDARG00000018149 | slc38a4 |
| ENSDARG00000034897 | rps10 | ENSDARG00000055388 | vtna |
| ENSDARG00000046098 | ebp | ENSDARG00000056587 | cyp2r1 |
| ENSDARG00000091503 | fads6 | ENSDARG00000086654 | cbln11 |
| ENSDARG00000026444 | apoob | ENSDARG00000022689 | itgb1b.2 |
| ENSDARG00000101348 | plppr5b | ENSDARG00000060120 | glyctk |
| ENSDARG00000078859 | g0s2 | ENSDARG00000041645 | sb:cb37 |
| ENSDARG00000019532 | fads2 | ENSDARG00000037421 | egr1 |
| ENSDARG00000052361 | il15 | ENSDARG00000091136 | zgc:174259 |
| ENSDARG00000030687 | phka2 | ENSDARG00000057498 | habp2 |
| ENSDARG00000052099 | agxta | ENSDARG00000079486 | fam20a |
| ENSDARG00000102341 | sugct | ENSDARG00000026322 | dhrs13a.1 |
| ENSDARG00000069439 | ptgdsa | ENSDARG00000019541 | gpt2l |
| ENSDARG00000040881 | hnrnph1 | ENSDARG00000053620 | ebi3 |
| ENSDARG00000038199 | cdab | ENSDARG00000076900 | prozb |
| ENSDARG00000054300 | dhrs1 | ENSDARG00000001818 | c3b.2 |
| ENSDARG00000062085 | tkfc | ENSDARG00000032098 | c8g |
| ENSDARG00000076056 | IYD | ENSDARG00000042684 | serpinc1 |
| ENSDARG00000086740 | phyh | ENSDARG00000019294 | cbln8 |
| ENSDARG00000087359 | c3a.2 | ENSDARG00000089331 | urahb |
| ENSDARG00000098588 | gchfr | ENSDARG00000078757 | si:ch211-212c13.8 |
| ENSDARG00000087657 | fasn | ENSDARG00000059049 | zgc:174904 |
| ENSDARG00000045568 | bcat1 | ENSDARG00000074191 | zgc:172253 |
| ENSDARG00000013721 | g6pca.2 | ENSDARG00000102945 | ccl39.2 |
| ENSDARG00000041205 | slc6a1l | ENSDARG00000016412 | agt |
| ENSDARG00000015575 | cyp3c1 | ENSDARG00000078918 | comtd1 |
| ENSDARG00000099787 | echdc3 | ENSDARG00000069292 | acot16 |
| ENSDARG00000045447 | slc35g2b | ENSDARG00000011521 | upb1 |
| ENSDARG00000021250 | slc25a48 | ENSDARG00000105265 | zgc:171534 |
| ENSDARG00000099558 | NPC1L1 | ENSDARG00000010312 | cp |
| ENSDARG00000029866 | slc6a14 | ENSDARG00000052207 | c3a.3 |
| ENSDARG00000061896 | slco2a1 | ENSDARG00000058053 | serping1 |
| ENSDARG00000110341 | hsd11b1lb | ENSDARG00000012694 | c3a.1 |
| ENSDARG00000099315 | nsdhl | ENSDARG00000043719 | c3a.6 |
| ENSDARG00000079946 | sqlea | ENSDARG00000016319 | c9 |
| ENSDARG00000038293 | zgc:103559 | ENSDARG00000006526 | fn1b |
| ENSDARG00000032553 | egln3 | ENSDARG00000055036 | itih3a |
| ENSDARG00000033413 | acot18 | ENSDARG00000020741 | fga |
| ENSDARG00000026764 | ahsg1 | ENSDARG00000023157 | cbln10 |
| ENSDARG00000091650 | igflr1 | ENSDARG00000010267 | dpydb |
| ENSDARG00000012194 | scp2a | ENSDARG00000100782 | F7 |
| ENSDARG00000038865 | acox3 | ENSDARG00000022372 | kng1 |
| ENSDARG00000003257 | zgc:101559 | ENSDARG00000039517 | c8b |
| ENSDARG00000067851 | lcn15 | ENSDARG00000103760 | cfhl2 |
| ENSDARG00000102722 | itih3b | ENSDARG00000008969 | fgb |
| ENSDARG00000042114 | bin1a | ENSDARG00000004904 | gys2 |
| ENSDARG00000052408 | mgat2 | ENSDARG00000021004 | c5 |
| ENSDARG00000076838 | apom | ENSDARG00000039516 | c8a |
| ENSDARG00000100406 | zgc:112265 | ENSDARG00000102175 | hamp |
| ENSDARG00000078416 | zeb2b | ENSDARG00000102456 | cfhl4 |
| ENSDARG00000017794 | sdr16c5b | ENSDARG00000053684 | aldob |
| ENSDARG00000041623 | mt2 | ENSDARG00000093079 | proca |
| ENSDARG00000099336 | mvda | ENSDARG00000036239 | gatm |
| ENSDARG00000086569 | zgc:172051 | ENSDARG00000037281 | fgg |
| ENSDARG00000089156 | egr3 | ENSDARG00000002197 | pygl |
| ENSDARG00000038258 | proca | ENSDARG00000057128 | hadhaa |
| ENSDARG00000036041 | f2 | ENSDARG00000027992 | hao2 |
| ENSDARG00000021208 | serpind1 | ENSDARG00000093569 | bckdhbl |
| ENSDARG00000062864 | gk5 | ENSDARG00000023111 | plg |
| ENSDARG00000053831 | vtnb | ENSDARG00000057035 | stoml3b |
| ENSDARG00000007127 | acat2 | ENSDARG00000042379 | zgc:103681 |
| ENSDARG00000017444 | hal | ENSDARG00000037873 | cyp3c3 |
| ENSDARG00000034862 | f7 | ENSDARG00000070810 | ccka |
| ENSDARG00000101762 | fah | ENSDARG00000090301 | usp48 |
| ENSDARG00000061274 | lss | ENSDARG00000063559 | pigx |
| ENSDARG00000090850 | serpina1l | ENSDARG00000031299 | dhrs11b |
| ENSDARG00000053973 | fetub | ENSDARG00000101629 | CABZ01079192.1 |
| ENSDARG00000037144 | cpb2 | ENSDARG00000091560 | slc6a19a.2 |
| ENSDARG00000028163 | prg4b | ENSDARG00000053858 | crip1 |
| ENSDARG00000004296 | ambp | ENSDARG00000054211 | st8sia7.1 |
| ENSDARG00000103308 | mst1 | ENSDARG00000099525 | si:ch1073-13h15.3 |
| ENSDARG00000098766 | pcxa | ENSDARG00000056719 | slc6a19b |
| ENSDARG00000008816 | glud1a | ENSDARG00000016081 | cldn15la |
| ENSDARG00000018643 | igf2a | ENSDARG00000102051 | mtbl |
| ENSDARG00000031046 | nr1h5 | ENSDARG00000095272 | htr7c |
| ENSDARG00000075827 | f7i | ENSDARG00000075151 | si:dkey-188i13.10 |
| ENSDARG00000030872 | cetp | ENSDARG00000079873 | SLC29A4 |
| ENSDARG00000095807 | hp | ENSDARG00000037425 | s100a10a |
| ENSDARG00000056314 | a2ml | ENSDARG00000067848 | nmrk2 |
| ENSDARG00000043279 | aqp12 | ENSDARG00000058332 | krt18a.2 |
| ENSDARG00000054319 | oxct1b | ENSDARG00000056026 | tprg1 |
| ENSDARG00000056196 | slc2a2 | ENSDARG00000100635 | chia.1 |
| ENSDARG00000002764 | miox | ENSDARG00000056108 | ndufa4 |
| ENSDARG00000087697 | lpl | ENSDARG00000029473 | gstm.2 |
| ENSDARG00000019986 | grhprb | ENSDARG00000055538 | avl9 |
| ENSDARG00000079727 | selenop2 | ENSDARG00000093078 | plscr3a |
| ENSDARG00000045516 | itih2 | ENSDARG00000078078 | lrfn4b |
| ENSDARG00000052734 | hmgcra | ENSDARG00000029795 | prxl2b |
| ENSDARG00000099200 | zgc:123103 | ENSDARG00000069549 | zgc:162396 |
| ENSDARG00000093068 | c3b.1 | ENSDARG00000089361 | wu:fb59d01 |
| ENSDARG00000074684 | mlsl | ENSDARG00000094112 | slc22a21 |
| ENSDARG00000026904 | cbln13 | ENSDARG00000057706 | si:ch211-137i24.10 |
| ENSDARG00000075614 | apoc4 | ENSDARG00000032639 | cd36 |
| ENSDARG00000061383 | serpinf2b | ENSDARG00000003902 | ctsl.1 |
| ENSDARG00000031616 | g6pca.1 | ENSDARG00000013871 | slc5a1 |
| ENSDARG00000007988 | masp2 | ENSDARG00000022183 | gsto1 |
| ENSDARG00000092155 | apoc2 | ENSDARG00000074698 | sdsl |
| ENSDARG00000069293 | ahsg2 | ENSDARG00000070480 | agr2 |
| ENSDARG00000093193 | chia.6 | ENSDARG00000003219 | bin2a |
| ENSDARG00000040890 | fdps | ENSDARG00000091624 | ugt2a7 |
| ENSDARG00000071592 | aqp8a.2 | ENSDARG00000019228 | mogat2 |
| ENSDARG00000095633 | si:ch211-133l5.7 | ENSDARG00000087867 | SLC2A13 |
| ENSDARG00000042293 | ca4b | ENSDARG00000058638 | si:ch211-71m22.1 |
| ENSDARG00000030980 | csrp1b | ENSDARG00000069244 | si:ch211-244b2.3 |
| ENSDARG00000104721 | si:dkey-203a12.9 | ENSDARG00000044528 | slc15a1b |
| ENSDARG00000097231 | ugt1b2 | ENSDARG00000076090 | jakmip1 |
| ENSDARG00000044566 | fabp6 | ENSDARG00000013056 | itpk1a |
| ENSDARG00000068088 | tcnba | ENSDARG00000079647 | muc13b |
| ENSDARG00000058327 | neu3.3 | ENSDARG00000017047 | slc27a4 |
| ENSDARG00000069018 | cyp7a1 | ENSDARG00000059729 | sec14l8 |
| ENSDARG00000008029 | mep1a.2 | ENSDARG00000100531 | abhd14b |
| ENSDARG00000018361 | sult1st3 | ENSDARG00000110439 | ap1s3a |
| ENSDARG00000041115 | cnfn | ENSDARG00000087402 | tpm1 |
| ENSDARG00000077688 | cpo | ENSDARG00000076265 | ano8a |
| ENSDARG00000003615 | slc26a3.2 | ENSDARG00000099116 | nagk |
| ENSDARG00000104213 | si:dkey-283b1.6 | ENSDARG00000030478 | zgc:66484 |
| ENSDARG00000098162 | zgc:77748 | ENSDARG00000099728 | zgc:56622 |
| ENSDARG00000042920 | sult3st3 | ENSDARG00000007302 | sh3gl3b |
| ENSDARG00000016918 | ace2 | ENSDARG00000097193 | si:dkey-28n18.9 |
| ENSDARG00000043729 | plac8.1 | ENSDARG00000101160 | apoa4a |
| ENSDARG00000005221 | desmb | ENSDARG00000090072 | CU462878.1 |
| ENSDARG00000068220 | mbl2 | ENSDARG00000093303 | ifitm1 |
| ENSDARG00000037551 | pm20d1.1 | ENSDARG00000011661 | twf1b |
| ENSDARG00000074556 | ganc | ENSDARG00000071377 | hsd11b1la |
| ENSDARG00000070775 | cyp2x9 | ENSDARG00000005704 | eps8l3b |
| ENSDARG00000103878 | anpepb | ENSDARG00000102744 | mgst3a |
| ENSDARG00000099185 | chia.2 | ENSDARG00000038154 | isca2 |
| ENSDARG00000076981 | zgc:198329 | ENSDARG00000057714 | cmah |
| ENSDARG00000101051 | ctsbb | ENSDARG00000005179 | dglucy |
| ENSDARG00000045180 | acta2 | ENSDARG00000042332 | plin2 |
| ENSDARG00000112419 | si:ch211-62a1.3 | ENSDARG00000098746 | dhrs13l1 |
| ENSDARG00000053625 | anxa2b | ENSDARG00000041108 | ctsh |
| ENSDARG00000093044 | si:ch211-161h7.5 | ENSDARG00000009939 | micu2 |
| ENSDARG00000068181 | dpep1 | ENSDARG00000057064 | enpep |
| ENSDARG00000061355 | aoc1 | ENSDARG00000056378 | ifi30 |
| ENSDARG00000003977 | zgc:153896 | ENSDARG00000005112 | cdh17 |
| ENSDARG00000054288 | zgc:63972 | ENSDARG00000100938 | hint2 |
| ENSDARG00000040898 | gpa33b | ENSDARG00000015566 | dnmt3ab |
| ENSDARG00000103019 | gstp2 | ENSDARG00000088116 | gstm.3 |
| ENSDARG00000103659 | bco1l | ENSDARG00000071087 | slc35b4 |
| ENSDARG00000079443 | vipb | ENSDARG00000099923 | zgc:154054 |
| ENSDARG00000070021 | cyp3c4 | ENSDARG00000037307 | gnpda1 |
| ENSDARG00000012388 | cox4i1l | ENSDARG00000053774 | alpi.2 |
| ENSDARG00000100955 | stk24a | ENSDARG00000025311 | cuzd1.2 |
| ENSDARG00000075159 | meltf | ENSDARG00000007040 | tmigd1 |
| ENSDARG00000040466 | vil1 | ENSDARG00000096990 | si:ch1073-340i21.2 |
| ENSDARG00000090185 | si:dkeyp-73b11.8 | ENSDARG00000097456 | coa3b |
| ENSDARG00000058462 | zgc:158846 | ENSDARG00000035832 | pyyb |
| ENSDARG00000099227 | galnt13 | ENSDARG00000100315 | slc15a1a |
| ENSDARG00000034321 | palmdb | ENSDARG00000105351 | si:ch211-113d11.6 |
| ENSDARG00000014916 | slc10a2 | ENSDARG00000054786 | faah2b |
| ENSDARG00000075800 | calml4a | ENSDARG00000089050 | cuzd1.1 |
| ENSDARG00000022509 | cox4i2 | ENSDARG00000041540 | sult1st2 |
| ENSDARG00000091609 | spink4 | ENSDARG00000012903 | slc34a2a |
| ENSDARG00000094730 | acbd7 | ENSDARG00000056877 | vamp2 |
| ENSDARG00000001870 | atp1a1a.4 | ENSDARG00000007743 | il15l |
| ENSDARG00000012829 | asah2 | ENSDARG00000018750 | npr1b |
| ENSDARG00000096445 | si:ch211-214p16.3 | ENSDARG00000101778 | ca5a |
| ENSDARG00000018621 | slc6a19a.1 | ENSDARG00000036291 | nucb2b |
| ENSDARG00000055416 | serpinb1 | ENSDARG00000036456 | anxa4 |
| ENSDARG00000020187 | GCA | ENSDARG00000029500 | rpl34 |
| ENSDARG00000024278 | adh8b | ENSDARG00000029177 | lnx2a |
| ENSDARG00000070966 | insl5a | ENSDARG00000101127 | map1lc3b |
| ENSDARG00000058226 | ak3 | ENSDARG00000062467 | stra6l |
| ENSDARG00000055656 | pdzd3b | ENSDARG00000007425 | apol1 |
| ENSDARG00000071306 | gip | ENSDARG00000021339 | cpa5 |
| ENSDARG00000078389 | ifi46 | ENSDARG00000041702 | rfx6 |
| ENSDARG00000039117 | tefa | ENSDARG00000027803 | sbds |
| ENSDARG00000040683 | MEP1B | ENSDARG00000035859 | angptl4 |
| ENSDARG00000102004 | apoea | ENSDARG00000020218 | amfra |
| ENSDARG00000039116 | nr5a5 | ENSDARG00000043168 | cela1.5 |
| ENSDARG00000059227 | fabp1b.1 | ENSDARG00000043175 | zgc:112266 |
| ENSDARG00000014556 | serpinb1l3 | ENSDARG00000010844 | kras |
| ENSDARG00000051876 | ush1c | ENSDARG00000054239 | ghrl |
| ENSDARG00000039832 | gsta.2 | ENSDARG00000030357 | amy2al1 |
| ENSDARG00000102482 | zgc:165573 | ENSDARG00000052110 | mboat4 |
| ENSDARG00000003795 | idh2 | ENSDARG00000056491 | ikzf5 |
| ENSDARG00000070427 | s100v1 | ENSDARG00000041110 | dnajc3a |
| ENSDARG00000045408 | tagln | ENSDARG00000013310 | map3k15 |
| ENSDARG00000033285 | gsto2 | ENSDARG00000060542 | lgr4 |
| ENSDARG00000092916 | mlnl | ENSDARG00000042590 | rbm19 |
| ENSDARG00000029011 | xpnpep1 | ENSDARG00000017235 | eif5a |
| ENSDARG00000068680 | ctrl | ENSDARG00000003270 | dhps |
| ENSDARG00000090428 | ctrb1 | ENSDARG00000040907 | gcgb |
| ENSDARG00000017490 | cel.1 | ENSDARG00000017445 | eif3i |
| ENSDARG00000013856 | amy2a | ENSDARG00000012269 | clcn1b |
| ENSDARG00000079274 | prss59.1 | ENSDARG00000101626 | arf6a |
| ENSDARG00000007276 | ela3l | ENSDARG00000058011 | arxa |
| ENSDARG00000017314 | cela1.6 | ENSDARG00000005191 | ahcy |
| ENSDARG00000026799 | suv39h1a | ENSDARG00000006963 | cse1l |
| ENSDARG00000100184 | ly97.2 | ENSDARG00000058953 | abcc4 |
| ENSDARG00000018263 | pdia2 | ENSDARG00000013931 | eif3m |
| ENSDARG00000011023 | pdlim3a | ENSDARG00000077530 | ahctf1 |
| ENSDARG00000043173 | cela1.3 | ENSDARG00000052928 | arf6b |
| ENSDARG00000100086 | adipoqb | ENSDARG00000054916 | eif4ebp3 |
| ENSDARG00000042993 | prss1 | ENSDARG00000091418 | snx17 |
| ENSDARG00000030915 | cpa1 | ENSDARG00000060211 | ankef1b |
| ENSDARG00000031438 | kcnj11l | ENSDARG00000100947 | mafk |
| ENSDARG00000043722 | cpa4 | ENSDARG00000079296 | gcga |
| ENSDARG00000100844 | cldn15lb | ENSDARG00000099291 | lsr |
| ENSDARG00000056765 | ela2l | ENSDARG00000013628 | cd164 |
| ENSDARG00000068144 | nmba | ENSDARG00000010146 | cpa2 |
| ENSDARG00000074697 | npdc1a | ENSDARG00000014479 | ptf1a |
| ENSDARG00000044813 | vps33b | ENSDARG00000019135 | ankef1a |
| ENSDARG00000017880 | kcnip3b | ENSDARG00000014190 | sst |
| ENSDARG00000092136 | cenpw | ENSDARG00000040747 | tm4sf4 |
| ENSDARG00000019566 | neurod1 | ENSDARG00000008931 | renbp |
| ENSDARG00000071699 | ids | ENSDARG00000036868 | trpl1 |
| ENSDARG00000086288 | scg3 | ENSDARG00000070047 | rgs4 |
| ENSDARG00000101606 | rims2a | ENSDARG00000079922 | klf4 |
| ENSDARG00000079862 | kl | ENSDARG00000002600 | pcsk1 |
| ENSDARG00000112049 | cldn15a | ENSDARG00000032126 | scg5 |
| ENSDARG00000008398 | cacna1c | ENSDARG00000057315 | rwdd3 |

**Supplementary Table S6. Regulators of endodermal organ development**

| **Gene** | **GeneName** | **Gene** | **GeneName** |
| --- | --- | --- | --- |
| ENSDARG00000021336 | pax4 | ENSDARG00000042725 | cebpb |
| ENSDARG00000044059 | lft2 | ENSDARG00000037397 | ssrp1a |
| ENSDARG00000116139 | cdx1b | ENSDARG00000034893 | rarab |
| ENSDARG00000057096 | ndr1 | ENSDARG00000014091 | osr1 |
| ENSDARG00000055630 | Foxh1 | ENSDARG00000056783 | raraa |
| ENSDARG00000011941 | bmpr2a | ENSDARG00000038867 | shhb |
| ENSDARG00000015906 | mxtx2 | ENSDARG00000006640 | Eomesa |
| ENSDARG00000007329 | tbx16 | ENSDARG00000103379 | pax6a |
| ENSDARG00000075113 | Nanog | ENSDARG00000101576 | Tbxta |
| ENSDARG00000055283 | id2a | ENSDARG00000098952 | gata4 |
| ENSDARG00000037514 | hdac3 | ENSDARG00000033172 | nr2f5 |
| ENSDARG00000071583 | gtf3ab | ENSDARG00000034518 | trdmt1 |
| ENSDARG00000055926 | foxi3a | ENSDARG00000042577 | batf3 |
| ENSDARG00000011235 | Otx2b | ENSDARG00000038990 | wnt3 |
| ENSDARG00000090656 | tomm20a | ENSDARG00000032234 | wnt2bb |
| ENSDARG00000014479 | ptf1a | ENSDARG00000007277 | myf5 |
| ENSDARG00000029263 | hoxb3a | ENSDARG00000100475 | fgf10b |
| ENSDARG00000068567 | shha | ENSDARG00000053479 | gdf6a |
| ENSDARG00000021494 | hnf4a | ENSDARG00000012788 | foxa3 |
| ENSDARG00000000767 | spi1b | ENSDARG00000103589 | gata6 |
| ENSDARG00000099880 | sp6 | ENSDARG00000055158 | prox1a |
| ENSDARG00000002707 | her11 | ENSDARG00000052511 | lin28b |
| ENSDARG00000010312 | cp | ENSDARG00000031420 | wt1a |
| ENSDARG00000052348 | smarca5 | ENSDARG00000012649 | Smad4 |
| ENSDARG00000070818 | pax7b | ENSDARG00000054632 | fli1a |
| ENSDARG00000094965 | nfil3-5 | ENSDARG00000029072 | klf6a |
| ENSDARG00000103409 | uhrf1 | ENSDARG00000105045 | bmpr1ab |
| ENSDARG00000002779 | pdx1 | ENSDARG00000077852 | samd11 |
| ENSDARG00000010192 | pax3a | ENSDARG00000006389 | smad2 |
| ENSDARG00000069640 | vegfc | ENSDARG00000004415 | TCF7L2 |
| ENSDARG00000053298 | Nkx2.2a | ENSDARG00000093420 | creb1b |
| ENSDARG00000027199 | smad1 | ENSDARG00000021389 | jag2b |
| ENSDARG00000013533 | hoxb4a | ENSDARG00000100591 | sox32 |
| ENSDARG00000019920 | lft1 | ENSDARG00000067673 | snapc4 |
| ENSDARG00000034117 | rarga | ENSDARG00000103554 | notch1a |
| ENSDARG00000017821 | gata5 | ENSDARG00000103554 | notch1a |
| ENSDARG00000005150 | tbx20 | ENSDARG00000062553 | bach1a |
| ENSDARG00000057633 | cxcr4a | ENSDARG00000074378 | junba |
| ENSDARG00000104100 | bmpr1ba | ENSDARG00000041430 | bmp2b |
| ENSDARG00000058822 | wnt3a | ENSDARG00000025641 | gli2a |
| ENSDARG00000003411 | foxa2 | ENSDARG00000077982 | elf3 |
| ENSDARG00000098231 | hhex | ENSDARG00000013168 | jag1b |
| ENSDARG00000091029 | phox2bb | ENSDARG00000030932 | fgf10a |
| ENSDARG00000036096 | smad3a | ENSDARG00000019732 | mtch2 |
| ENSDARG00000044774 | Pou5f3 | ENSDARG00000056023 | hoxb9a |
| ENSDARG00000017696 | utp25 | ENSDARG00000068401 | yap1 |
| ENSDARG00000102138 | foxa1 | ENSDARG00000037238 | smad5 |
| ENSDARG00000037677 | fgf24 | ENSDARG00000019728 | bmpr1aa |
| ENSDARG00000014181 | foxp1b | ENSDARG00000011027 | fgfr1a |
| ENSDARG00000101199 | rbp4 | ENSDARG00000067719 | wwtr1 |
| ENSDARG00000103308 | mst1 | ENSDARG00000030756 | dnmt1 |
| ENSDARG00000041959 | cxcr4b | ENSDARG00000005315 | Celf1 |
| ENSDARG00000017121 | mafba | ENSDARG00000030110 | myod1 |
| ENSDARG00000116511 | npas4l | ENSDARG00000104773 | junbb |
| ENSDARG00000018902 | pes | ENSDARG00000014246 | jag2a |
| ENSDARG00000102742 | bmpr1bb | ENSDARG00000015427 | hdac1 |
| ENSDARG00000008188 | sf1 | ENSDARG00000019995 | bmp4 |
| ENSDARG00000045146 | tomm22 | ENSDARG00000070913 | Sox2 |
| ENSDARG00000007823 | atf3 | ENSDARG00000036074 | cebpa |
| ENSDARG00000020057 | bmpr2b | ENSDARG00000043130 | notch2 |

**Supplementary Table S7. List of key resources and notes**

| **REAGENT or RESOURCE** | **SOURCE** | **IDENTIFIER** |
| --- | --- | --- |
| **Antibodies** | | |
| Rabbit monoclonal Wdr5 antibody | Huabio | Cat#ET1705-60 |
| Rabbit monoclonal Wdr5 antibody | Abcam | Cat#ab178410 |
| Rabbit polyclonal H4K16ac antibody | Merck | Cat#07-329 |
| Rabbit polyclonal H3K4me3 antibody | Abcam | Cat#ab8580 |
| Rabbit polyclonal beta-Catenin antibody | GeneTex | Cat#GTX101435 |
| Rabbit monoclonal beta-Actin antibody | Abclonal | Cat#AC026 |
| Mouse monoclonal HA-tag antibody | Abclonal | Cat#AE008 |
| Mouse monoclonal Bhmt antibody | Huabio | Clone#D5G5 |
| Mouse monoclonal P53 antibody | Huabio | Clone#A7C10 |
| Rabbit polyclonal p-Histone H3 | Santa Cruz | Cat#SC-8656-R |
| Rabbit monoclonal Histone3 antibody | CST | Cat#4499 |
| Mouse monoclonal Flag antibody | Huabio | Cat#HA601080 |
| HA-tag mouse antibody(Agarose conjugated) | Abmart | Cat#M20013M |
| **Critical Commercial Assays** | | |
| BCIP/NBT | Beyotime | Cat#C3206 |
| DIG RNA Labeling Mix,10x conc. | Roche | Cat#11277065910 |
| DIG RNA Labeling Mix,10x conc. | Roche | Cat#11277073910 |
| EasySee Western Blot Kit | Trans | Cat#DW101-02 |
| In Situ Cell Death Det. Kit TMR red | Roche | Cat#12156792910 |
| Maxi ECL Substrate | Sunky | Cat#61804 |
| PCR Cleanup Kit | Axygen | Cat#156 |
| Plasmid Miniprep Kit | Axygen | Cat#155 |
| HiScribe™ T7 High Yield RNA Synthesis Kit | New England Biolabs | Cat#E2040S |
| mMESSAGE mMACHINE™ SP6 | ThermoFisher | Cat#AM1340 |
| Salinomycin sodium salt | MCE | Cat#HY-17439 |
| **Experimental Models: Zebrafish** | | |
| Zebrafish: Tübingen | Singapore | N/A |
| Zebrafish: wdr5^△10^ | This paper | N/A |
| Zebrafish: apc^WT/△8^ | This paper | N/A |
| Zebrafish: zgc:171740^+10/+10^ | This paper | N/A |
