# Supplementary material for: Wdr5-mediated H3K4me3 coordinately regulates cell differentiation, proliferation termination, and survival in digestive organogenesis
Source: Cell Death Discov. 2023 Jul 5;9:227. doi: 10.1038/s41420-023-01529-4 (PMC10323123; doi:10.1038/s41420-023-01529-4)
Supplement: Supplementary file 10 — author contribution [file 41420_2023_1529_MOESM10_ESM.pdf]

DECLARATION OF CONTRIBUTIONS TO ARTICLE

ADMC

Manuscript Number: CDDISCOVERY-23-0577R

Journal Name: *Cell Death Discovery* (the 'Journal')

Proposed Title of the Contribution:

Wdr5-mediated H3K4me3 coordinately regulates cell differentiation, proliferation termination and survival in digestive organogenesis (the 'Contribution)

Author(s):

Zhe Zhang, Chun Yang, Zixu Wang, Liwei Guo, Yongpan Xu, Ce Gao, Yonghua Sun, Zhenhai Zhang, Jinrong Peng, Minjie Hu, Li Jan Lo, Zhipeng Ma, and Jun Chen, (the 'Authors')

For all *CDDis*covery articles, each person named as an author in the published version must be able to show he or she has contributed substantially to the article.

Authorship credit should be based on 1) substantial contributions to conception and design, acquisition of data, or analysis and interpretation of data; 2) drafting the article or revising it critically for important intellectual content; and 3) final approval of the version to be published. Authors should meet conditions 1, 2 and 3.

Any person who cannot be shown to have made a substantial contribution to the article cannot be listed as an author in the final version. The name of any person who is deemed to have made a minor contribution can, however, appear in the Acknowledgments section of the article.

Please complete the table below to indicate the contributions of all named authors to the manuscript.

Author Full Name:	Specification of Contribution to the Manuscript:
Zhe Zhang	conception and design, acquisition of data, analysis and interpretation of data, drafting article
Chun Yang	acquisition of data
Zixu Wang	acquisition of data
Liwei Guo	acquisition of data
Yongpan Xu	acquisition of data
Ce Gao	acquisition of data
Yonghua Sun	revising it critically for important intellectual content
Zhenhai Zhang	revising it critically for important intellectual content
Jinrong Peng	revising it critically for important intellectual content
Minjie Hu	acquisition of data
Li Jan Lo	laboratory management
Zhipeng Ma	acquisition of data
Jun Chen	conception and design, acquisition of data, analysis and interpretation of data, drafting article

Please complete the table below to indicate the contributions of all named authors to the figures.

Figure 1:

Zhe Zhang, Liwei Guo, Zhipeng Ma

Figure 2:

Zhe Zhang, Chun Yang, Minjie Hu, Ce Gao

Figure 3:

Zhe Zhang, Yongpan Xu

Figure 4:

Zhe Zhang, Zixu Wang

Figure 5:

Zhe Zhang, Zhipeng Ma

Figure 6:

Zhe Zhang, Zixu Wang

Figure 7:

Zhe Zhang, Chun Yang

Signed for and on behalf of the Author(s):	Print Name:	Date:
Junchen	Jun Chen	JUN, 5, 2023
