# Supplementary material for: Wdr5-mediated H3K4me3 coordinately regulates cell differentiation, proliferation termination, and survival in digestive organogenesis
Source: Cell Death Discov. 2023 Jul 5;9:227. doi: 10.1038/s41420-023-01529-4 (PMC10323123; doi:10.1038/s41420-023-01529-4)
Supplement: Supplementary file 11 — Original Data File [file 41420_2023_1529_MOESM11_ESM.pdf]

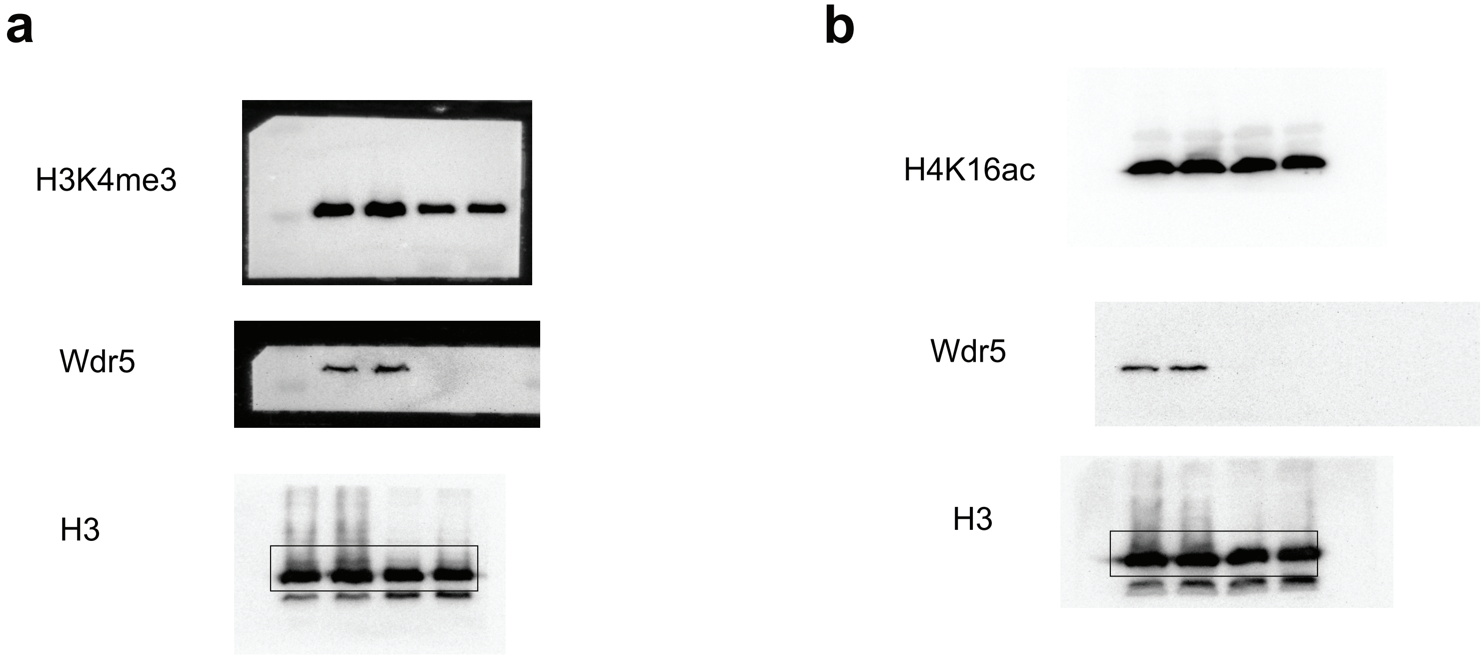

Figure. 2

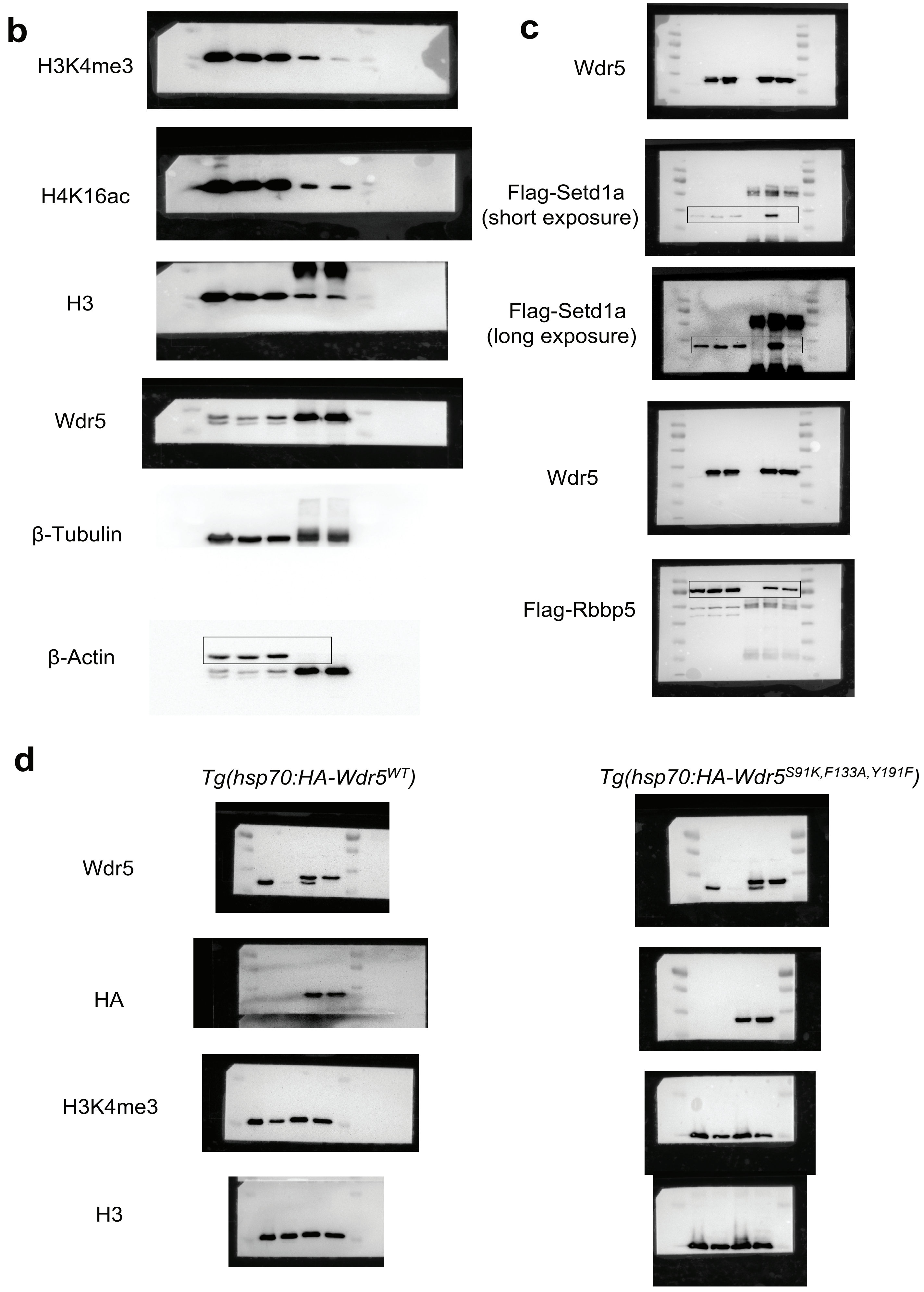
b
H3K4me3
H4K16ac
H3
Wdr5
β-Tubulin
β-Actin
c
Wdr5
Flag-Setd1a
(short exposure)
Flag-Setd1a
(long exposure)
Wdr5
Flag-Rbbp5
d
Tg(hsp70:HA-Wdr5^WT)
Tg(hsp70:HA-Wdr5^S91K,F133A,Y191F)
Wdr5
HA
H3K4me3
H3

Figure. 3

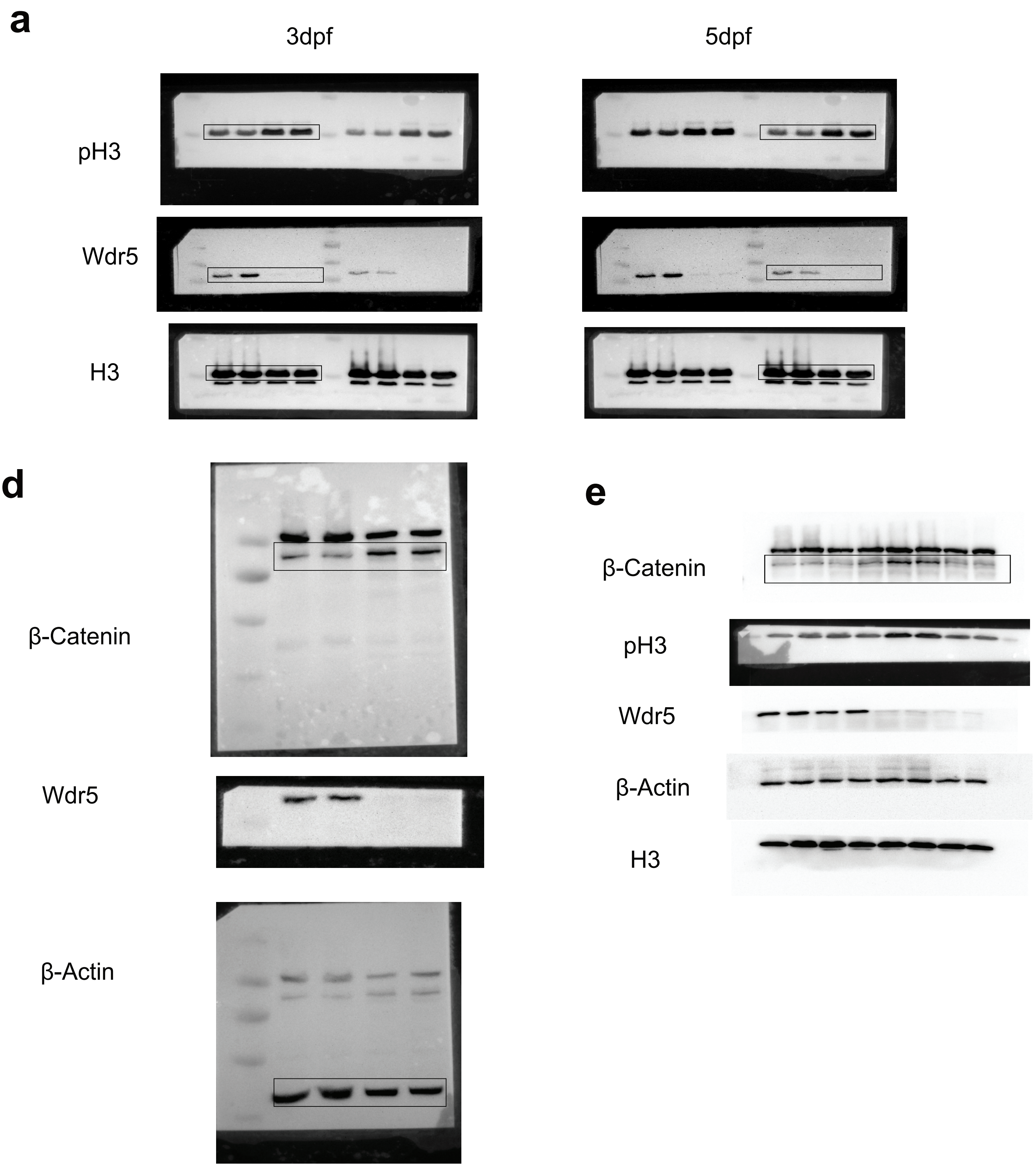
a
3dpf
5dpf
pH3
Wdr5
H3
d
β-Catenin
Wdr5
β-Actin
e
β-Catenin
pH3
Wdr5
β-Actin
H3

Figure. 4

c

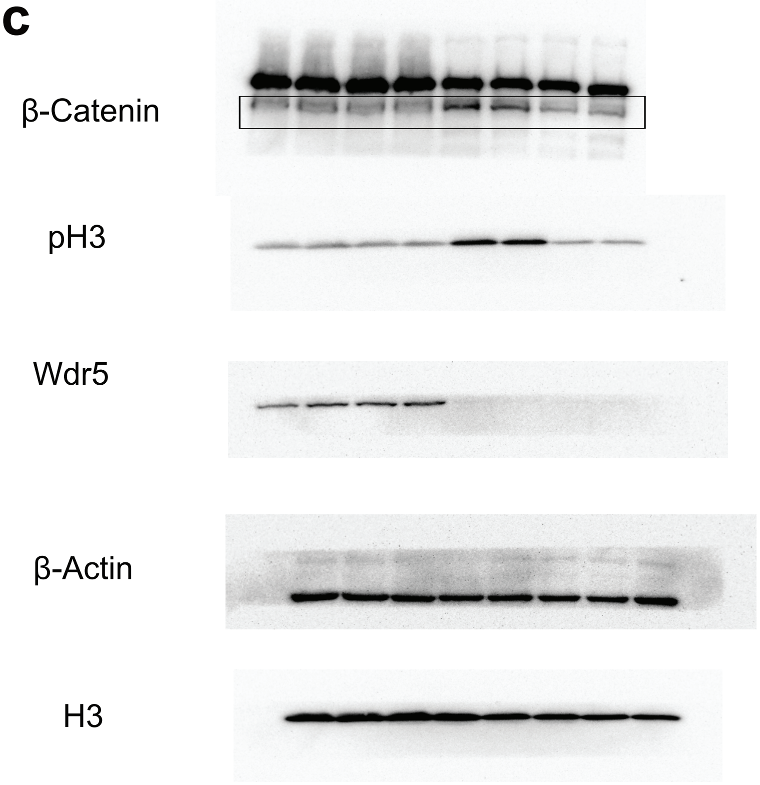

e

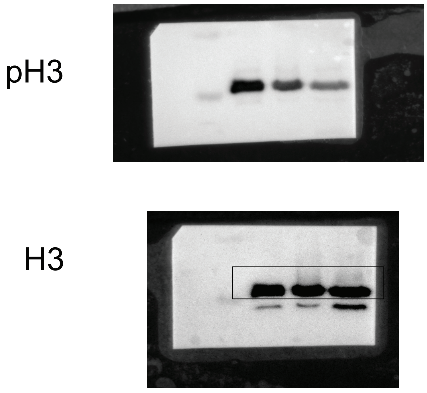

f

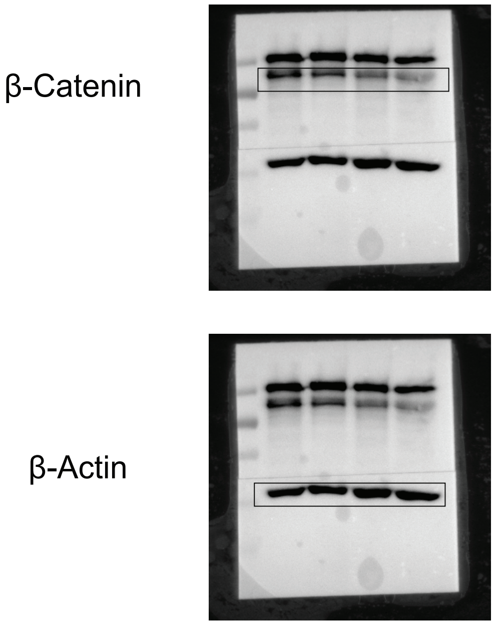

Figure. 5

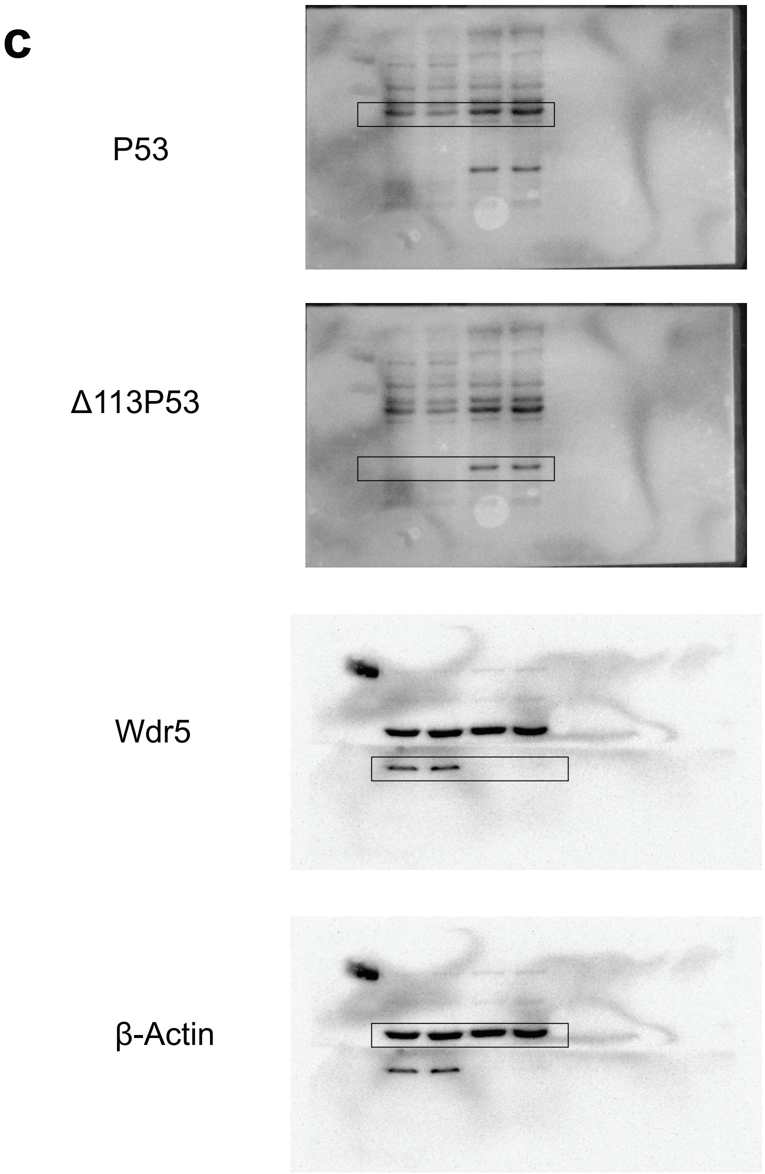

Figure. 6

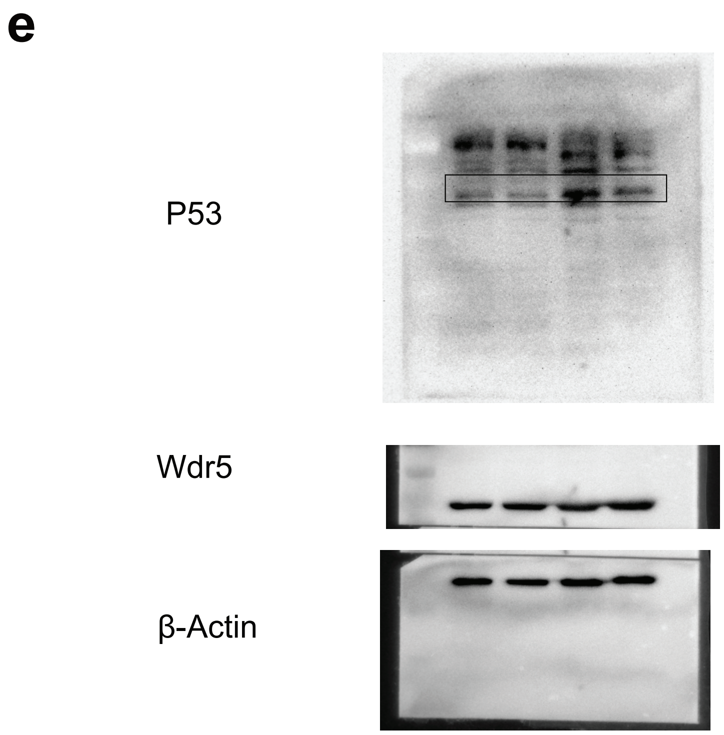

Figure. 7

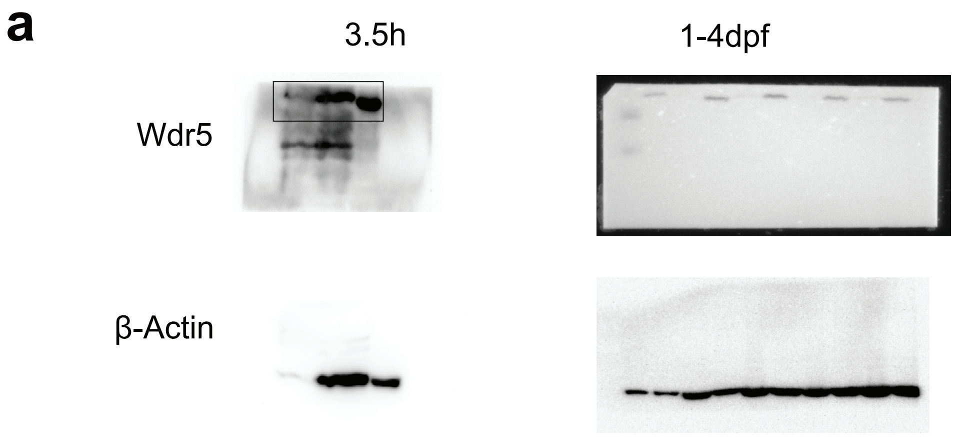

Supplementary Fig.1

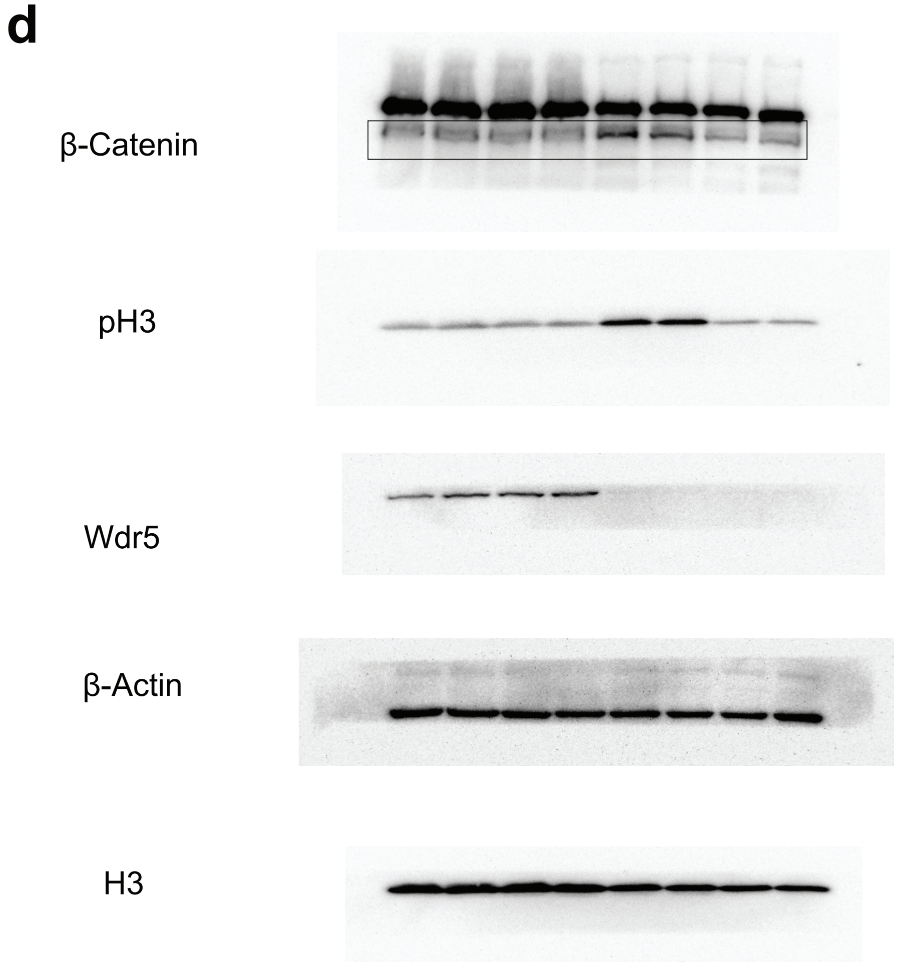

Supplementary Fig.6

f

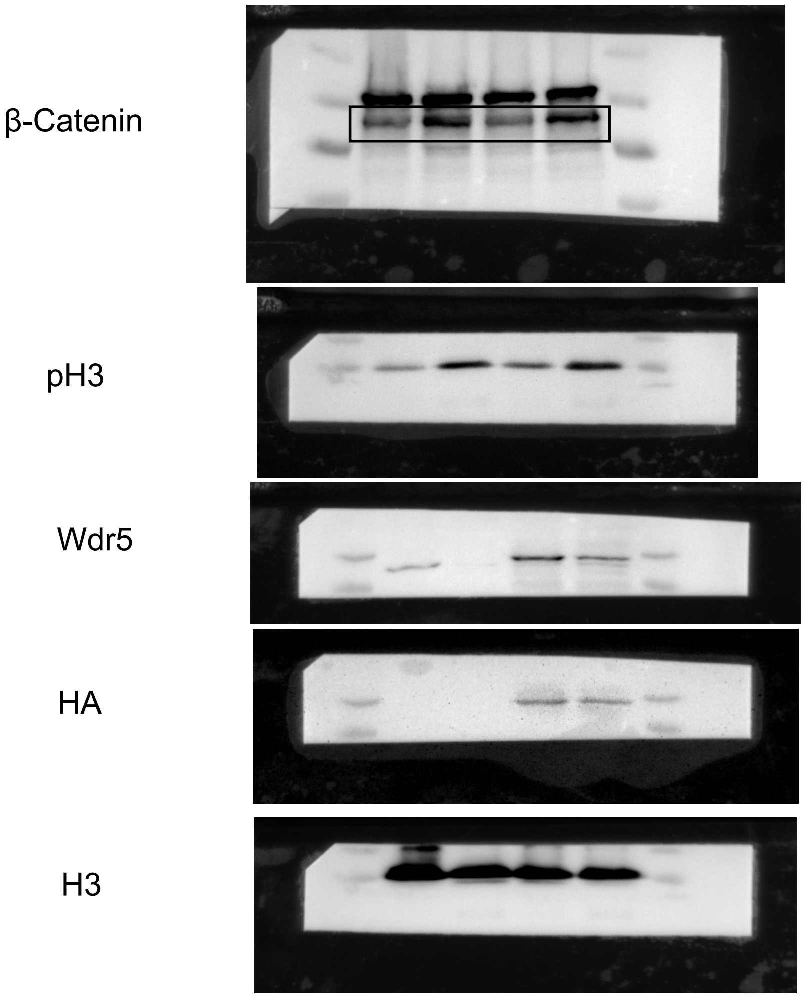

Supplementary Fig.7

d

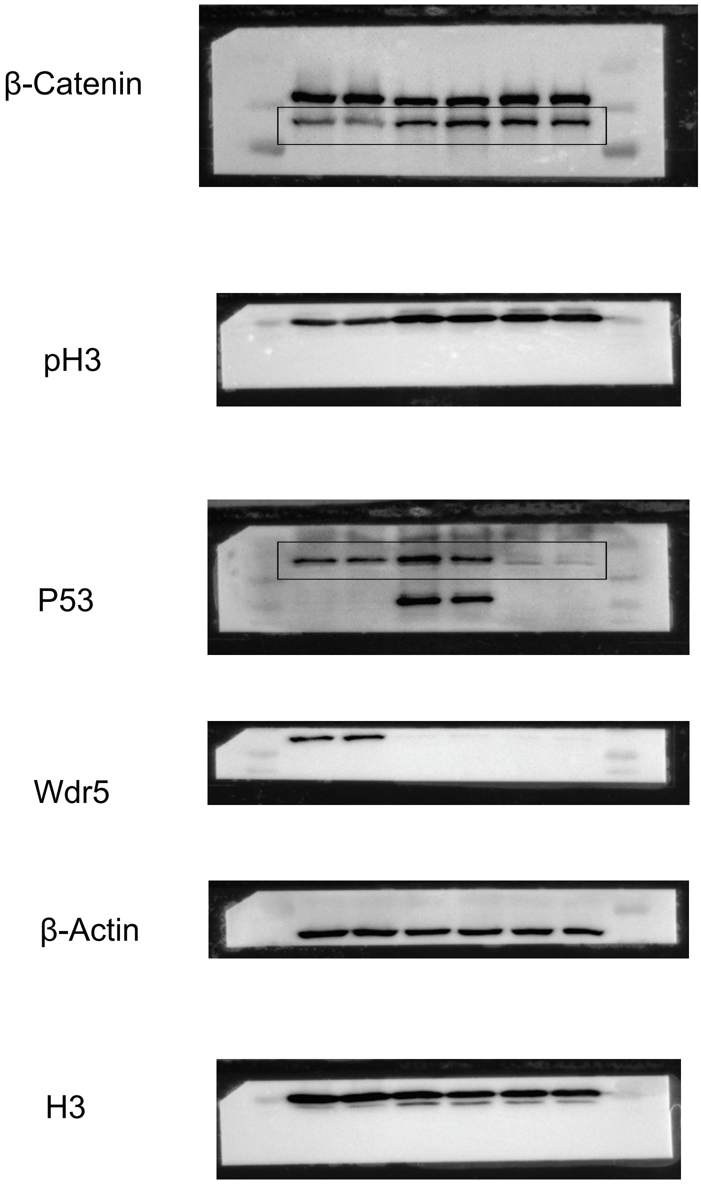

Supplementary Fig.8
